# Mutation-class dependent signatures outweigh disease-associated processes in cystic fibrosis cells

**DOI:** 10.1186/s13578-023-00975-y

**Published:** 2023-02-09

**Authors:** Lúcia Santos, Rui Nascimento, Aires Duarte, Violeta Railean, Margarida D. Amaral, Patrick T. Harrison, Margarida Gama-Carvalho, Carlos M. Farinha

**Affiliations:** 1 BioISI – Instituto de Biossistemas e Ciências Integrativas, Faculdade de Ciências, Universidade de Lisboa, 1749-016 Lisbon, Portugal; 2 Department of Physiology, University College Cork, Cork, T12 K8AF Ireland

**Keywords:** Cystic fibrosis, Disease signatures, Isogenic cell lines, Proteomics, Transcriptomics

## Abstract

**Background:**

The phenotypic heterogeneity observed in Cystic Fibrosis (CF) patients suggests the involvement of other genes, besides *CFTR*. Here, we combined transcriptome and proteome analysis to understand the global gene expression patterns associated with five prototypical *CFTR* mutations.

**Results:**

Evaluation of differentially expressed genes and proteins unveiled common and mutation-specific changes revealing functional signatures that are much more associated with the specific molecular defects associated with each mutation than to the CFTR loss-of-function phenotype. The combination of both datasets revealed that mutation-specific detected translated-transcripts (Dtt) have a high level of consistency.

**Conclusions:**

This is the first combined transcriptomic and proteomic study focusing on prototypical *CFTR* mutations. Analysis of Dtt provides novel insight into the pathophysiology of CF, and the mechanisms through which each mutation class causes disease and will likely contribute to the identification of new therapeutic targets and/or biomarkers for CF.

**Supplementary Information:**

The online version contains supplementary material available at 10.1186/s13578-023-00975-y.

## Introduction

Cystic fibrosis (CF) is the most common life-threatening autosomal recessive disease among the Caucasian population [1]. The disease is caused by mutations in the *Cystic Fibrosis Transmembrane Conductance Regulator (CFTR)* gene that encodes a chloride and bicarbonate channel expressed on the apical surface of the epithelial cells of multiple organs, including the lung, pancreas, and colon [1]. In the lung, where the most severe symptoms occur, CFTR impairment leads to an increase of mucus viscosity and compromised mucociliary clearance that promote chronic infection and inflammation, ultimately leading to respiratory failure [1].

To date, more than 2100 *CFTR* genetic variants have been reported and organized into seven classes (Class I-VII) according to their effect on CFTR protein [1, 2]. Class I mutations severely impair protein production and include mostly nonsense mutations, which cause premature stop codons and consequently degradation of mRNA by nonsense-mediated decay. Class II mutations cause protein misfolding and retention in the endoplasmic reticulum leading to premature degradation of CFTR which prevents its trafficking to the cell membrane. Class III mutations impair CFTR channel gating, while class IV mutations cause a reduction in CFTR channel conductance. Class V mutations lead to a decrease in the levels of normal CFTR, often because of alternative splicing events. Class VI mutations lead to a destabilization of CFTR at the cell surface, either by increasing endocytosis and/or decreasing recycling. Finally, class VII mutations result in impaired CFTR mRNA production, usually due to large deletions in the *CFTR* gene. Even though the frequency of *CFTR* mutation classes varies between countries, F508del, a class II mutation, is by far the most common; class I and III then account for most of the other mutations [3].

Although CF is recognized as a monogenic disease, there is considerable heterogeneity in its clinical outcomes [4]. The diversity of *CFTR* mutations can, to a large extent, explain the different phenotype severity. Technological advances in recent years have allowed the application of the so-called “omics” approaches to understanding CF. Identification of CF genetic modifiers has relied mainly on genome-wide association studies (GWAS). Transcriptomics, especially based on the use of microarrays, has been used to assess global patterns in gene expression, most of the time comparing wild-type samples with those bearing F508del, either in cell lines or in samples derived from individuals with CF [5–11]. Although microarray technology has had a significant level of success, it is limited by the transcripts targeted by the assay [12]. This problem was overcome through the development of massive parallel sequencing approaches and, in particular, their application to transcriptome analysis (RNA-seq). Although established more than a decade ago, the use of the RNA-Seq in the CF field is still limited to a couple of reports, e.g., assessing changes in the “blood” transcriptome to evaluate response to novel modulators or identify genetic modifiers [12, 13].

Proteomics has also been extensively used to better understand CF disease mechanisms, through the assessment of either the full proteome or the interactome in WT versus F508del samples, and in some cases applied to a few other mutations [14, 15]. Recent reports revisited the proteome using state-of-the-art proteomic methodologies in bronchial cells, either from stable lines expressing F508del-CFTR or primary cultures obtained from lung explants [16].

The use of omics approaches to characterize either transcript or protein levels can assist in the identification of which biological processes are different between diseased and healthy states, being useful for both the identification of markers of the disease process and of possible drug targets [17]. Integration of the different omics data types can overcome the eventual limitations of each individual approach and assist in the prioritization of critical causative changes leading to disease while providing deeper insights to support the development of alternative therapeutic approaches [17]. Indeed, although changes in the abundance of an mRNA and its encoded protein tend to display a reasonable correlation, the complex regulation underlying gene expression processes imply that transcriptomic and proteomic profiles hold non-redundant, complementary levels of information that can reveal relevant biological phenomena underlying disease pathophysiology [18].

Here, we present for the first time a combined transcriptomics and proteomics study in human bronchial epithelial cell lines CRISPR-engineered to express CFTR bearing five different *CFTR* in homozygosity from the endogenous promoter. Our study is the first to combine these two global characterization techniques to gain insight into CF, and it is the first to do so for an extended set of disease-causing mutations and in cells with endogenous expression of CFTR (eliminating the pitfalls associated with viral vector-based overexpression). Identification of differentially expressed genes and proteins, many of which fall into enriched gene ontology (GO) groups relevant to CF, including immune response, signalling pathways, cell differentiation, and actin cytoskeleton organization, showed a very good correlation between the two data sets. The validation of selected genes/proteins and the identification of general and mutation-specific differentially expressed genes and proteins provides novel insight into how different mutations (and mutation classes) cause CF and suggest novel potential therapeutic targets for the treatment of CF.

## Materials and methods

### 16HBE14o- gene-edited cell lines

16HBE14o- Human Bronchial Epithelial cells, expressing WT-CFTR, were obtained from Children’s Hospital Oakland Research Institute, UCSF, USA [19]. CFF-16HBE gene-edited cell lines with the following genotypes—G542X, F508del, N1303K, and G551D, W1282X and Y122X—were obtained from the Cystic Fibrosis Foundation (CFF) [20]. Cells were grown in Minimum Essential Medium (MEM, Sigma) supplemented with 10% (v/v) of Foetal Bovine Serum (FBS, Sigma) and 1% (v/v) of L-Glutamine (Sigma) in a 37 °C, 5% CO_2_ humidified incubator. Culture plates and flasks were coated by incubating a coating solution [Dulbecco’s Phosphate Buffered Saline (DPBS, Sigma) and 10 µL/mL of PureCol^®^ Solution (Advanced BioMatrix)] at 37 °C/5% CO_2_ for at least 2 h. Two additional cell lines carrying I1234V- and I507del-CFTR mutations were developed using the same conditions as described for the cell lines above. Briefly, the 100 µM crRNA and tracrRNA stocks were annealed in equimolar concentration to a final duplex concentration of 44 µM and incubated at 95 °C for 5 min. To form the RNP complex, the previously assembled crRNA:tracrRNA was mixed with Cas9 protein at a 1:1.2 M ratio and incubated at room temperature for 20 min. Cells were electroporated with the Neon™ Transfection System (Thermo Fisher Scientific) in the presence of the ssODN HDR template.

### CF subjects and ethical approval

Nasal brushing samples were obtained from one healthy donor and two CF individuals homozygous for the following mutations in the CFTR gene—F508del and N1303K. Informed consent was obtained from all the subjects and the study was conducted according to the guidelines of the Declaration of Helsinki and approved by the Ethics Committee of Hospital de Santa Maria (DIRCLN-16JUL2014-211).

### Primary human nasal epithelial (pHNE) cell cultures

Primary human nasal epithelial cells (pHNE) were isolated and cultured as previously described [21]. After isolation, cells were grown on a 6-well plate coated with collagen type I (30 μg/ml, Advanced BioMatrix) at a density of 500,000 cells/mL. After reaching 90–100% confluency, cells were seeded in triplicate (n = 3) in 12-well plates from which total RNA and protein were isolated as described below.

### RNA library preparation

Total RNA was isolated in triplicate (n = 3) from 16HBE14o- WT-CFTR and the five different gene-edited cell lines grown on 6-well plates with the NZY Total RNA Isolation kit (NZYTech) according to the manufacturer’s instructions. After measuring RNA integrity number (RIN) and concentration (≥ 7 and 1 µg, respectively), cDNA sequencing libraries were prepared using Stranded mRNA Library Preparation Kit according to manufacturer’s instructions and sequenced on Illumina Novaseq platform with paired-end 150 bp long reads (acquired as a service to STAB Vida).

### RNA-Seq data analysis

Following quality assessment using FastQC version 0.11.5 (https://www.bioinformaticshttps://www.bioinformaticsbabraham.ac.uk/projects/fastqc/), Cutadapt was used to remove sequencing adaptors and trim the first 10 nucleotides [22]. The trimmed data was then filtered using in-house perl scripts in order to remove reads with unknown nucleotides, homopolymers with length ≥ 50 nt or an average Phred score < 30 [23]. The remaining reads were aligned to the Genome Reference Consortium Human Build 38 (GRCh38) using the STAR aligner version 2.5.0 with the following options: –outFilterType BySJout –alignSJoverhangMin 8 –alignSJDBoverhangMin 5 –alignIntronMax 100000 –outSAMtype BAM SortedByCoordinate –twopassMode Basic –outFilterScoreMinOverLread 0 –outFilterMatchNminOverLread 0 –outFilterMatchNmin 0 –outFilterMultimapNmax 1 –limitBAMsortRAM 10000000000 –quantMode GeneCounts [24, 25]. Gene counts were determined using the htseq-count function from HTseq (version 0.9.1) in union mode and discarding low-quality score alignments (–a 10), using the Ensembl GRCh38.98 genome annotation.

DEA for RNA-Seq gene counts was performed with the limma Bioconductor package using the voom method to convert the read-counts to log2-cpm, with associated weights, for linear modelling [26, 27]. Samples in each group were treated as biological replicates and only genes that passed the significance cut-off were treated as DEGs. In this study, we used a p-value (FDR corrected p-value) less than 0.05 as the significance cut-off to identify DEGs.

### Transcriptomics data validation through RT-qPCR

To validate the RNA-Seq data, ten DEGs were selected for analysis by qRT-PCR. The same samples used for RNA sequencing were used for cDNA synthesis using M-MuLV Reverse Transcriptase (NZYTech) following the manufacturer’s instructions. Primers were designed using the NCBI Primer-BLAST online tool and were obtained from STAB VIDA (Additional file 1: Table S1). Specific products were amplified using Evagreen SsoFast PCR reagent (Bio-Rad) on the CFX96 Touch real-time PCR detection system (Bio-Rad). The fold difference in gene expression was calculated by the mathematical Eq. 2^−ΔΔCT^ and the GAPDH gene was used as an internal control.

### Protein extraction and sample preparation for mass spectrometry analysis

Total protein was extracted in triplicate (n = 3) from 16HBE14o- WT-CFTR and the five different gene-edited cell lines grown on 6-well plates. Cells were washed twice with 1 × PBS and solubilized in a lysis buffer containing 1.5% (w/v) Sodium-dodecyl-sulphate (SDS), 10% (v/v) glycerol, 0.5 mM dithiothreitol (DTT), 31.25 mM Tris (pH 6.8) and protease inhibitor cocktail (Roche). DNA was sheared by both 5U benzonase nuclease treatment (Sigma Aldrich) and using a 22G needle. Protein concentration was assessed with the Bradford assay.

One hundred micrograms of each sample were run on a 4–12% Bis–Tris gel, under denaturing conditions, for 5 min at 200 V with MES Running Buffer (NuPAGE, Invitrogen™) and stained with Instant Blue (Sigma). The whole run was cut into small pieces, distained with 50% acetonitrile (Optima^®^LC/MS grade, Fisher Scientific), and dehydrated with acetonitrile. The proteins were reduced with 10 mM dithiothreitol for 45 min at 56 °C (BioUltra, Sigma), alkylated with 55 mM iodoacetamide for 30 min at 20 °C in the dark (BioUltra, Sigma), and digested with 10 ng/µl trypsin overnight at 37 °C (Sequencing Grade Modified Trypsin V5111, Promega) in 50 mM ammonium bicarbonate (BioUltra, Sigma). The tryptic peptides were extracted from the gel with acetonitrile in an ultrasound bath (VWR), dried on a speed vac (ThermoSavant™ Scientific), resuspended in 5% formic acid (Optima LC/MS grade, Fisher Scientific) to perform peptide clean-up using two OMIX C18 100 µl pipette tip microcolumns (Agilent Technologies) and dried again. All the proteomics analysis were acquired as a service to the Mass Spectrometry Unit at Institute for Experimental Biology and Technology (iBET).

### Information-dependent acquisition (IDA) runs to generate the spectral library

Nano-liquid chromatography-tandem mass spectrometry (nanoLC-MS/MS) analysis was performed on an ekspert™ NanoLC 425 cHiPLC^®^ system coupled with a TripleTOF® 6600 with a NanoSpray® III source (Sciex). Peptides were separated through reversed-phase chromatography (RP-LC) in a trap-and-elute mode. Trapping was performed at 2 µl/min on a Nano cHiPLC Trap column (Sciex 200 µm × 0.5 mm, ChromXP C18-CL, 3 µm, 120 Å) with 100% A for 10 min. The separation was performed at 300 nl/min, on a Nano cHiPLC column (Sciex 75 µm × 15 cm, ChromXP C18-CL, 3 µm, 120 Å). The gradient was as follows: 0–1 min, 5% B (0.1% formic acid in acetonitrile, Fisher Chemicals, Geel, Belgium); 1–91 min, 5–30% B; 91–93 min, 30–80% B; 93–108 min, 80% B; 108–110 min, 80–5% B; 110–127 min, 5% B.

Peptides were sprayed into the MS through an uncoated fused-silica PicoTip^™^ emitter (360 µm O.D., 20 µm I.D., 10 ± 1.0 µm tip I.D., New Objective, Oullins, France). The source parameters were set as follows: 15 GS1, 0 GS2, 30 CUR, 2.5 keV ISVF, and 100 °C IHT. An information-dependent acquisition (IDA) method was set with a TOF–MS survey scan of 400–2000 m/z. The 50 most intense precursors were selected for subsequent fragmentation and the MS/MS were acquired in high sensitivity mode for 40 ms.

The raw files were subjected to database search in unison using ProteinPilot software v. 5.0 (Sciex, Framingham, US) with the Paragon algorithm to generate the Spectral Library. A UniProt database (20413 entries, accessed on 08/01/2021) containing the sequences of the proteins from Homo sapiens (Taxon ID: 9606) was used. The following search parameters were set: Iodoacetamide, as Cys alkylation; Trypsin, as digestion; TripleTOF 6600, as the Instrument; Gel-based ID, as Special factors; biological modifications, as ID focus; thorough, as search effort; and an FDR analysis enabled. Only the proteins with < 1% FDR were considered.

### Protein quantification by SWATH-MS

Three technical replicates from each biological replicate (n = 3) of each condition (n = 6) were analysed by sequential window acquisition of all theoretical fragment ion spectra (SWATH)-MS, using the instrument setup described for the IDA runs. The mass spectrometer was set to operate in cyclic data-independent acquisition (DIA), similar to the previously established method [28]. SWATH-MS data were acquired in SWATH acquisition mode using a set of 64 overlapping variable SWATH windows covering the precursor mass range of 400–1,400 m/z. The variable SWATH windows were calculated using the SWATH Variable Window Calculator V1.0 (Sciex, Framingham, US) based on a reference sample (IDA Wt_10ul_1.wiff). At the beginning of each cycle, a 20 ms survey scan (400-2,000 m/z) was acquired, and the subsequent SWATH windows were collected from 400 to 2000 m/z for 50 ms, resulting in a cycle time of 3.27 s. The collision energy for each window was set using rolling collision energy, and the collision energy spread was set to 5.

Data processing was performed using a SWATH processing plug-in for PeakView 2.2 (Sciex, Framingham, MA USA). First, the spectral library was imported with the maximum number of proteins to import set to 986, corresponding to a protein FDR < 1% Global FDR from fit and an unused protein score > 2.000. To calculate the false discovery rate (FDR) threshold, the MS/MS spectra were searched against a decoy database. Shared peptides were set not to be imported. Next, the RT calibration was performed by selecting peptides that covered the entire LC gradient. The calibration curve was manually inspected before proceeding with the RT calibration. Once the RT calibration was performed, data were processed using the following criteria: Number of peptides per protein: 6; Number of transitions per peptide: 6; Peptide confidence threshold: 98% (corresponding to a peptide FDR < 1% Global FDR from fit); False discovery rate threshold: 1%; Exclude modified peptides: No; Fix rank: No; XIC extraction window: 10 min; XIC width: 20 ppm. Manual inspection was performed for random peptides to check the quality of the data before data processing. Data were directly exported to Markerview 1.3.1 (Sciex, Framingham, MA USA) and normalized using total area sums to obtain the final quantification values. MarkerView was also used to perform the PCA and t-test statistical tests.

### Gene ontology (GO) enrichment analysis

Functional enrichment of DEGs and DEPs was performed with the GOfuncR package version 1.14.0 using the *Homo sapiens* annotation package “org.Hs.eg.db”. The core function goenrich was run with default parameters (hypergeometric test, 1000 randomizations) considering all the detected transcripts or proteins as background [29]. Go terms were considered significantly with an FWER < 0.05 for the DEG dataset and a p-value < 0.01 for the DEP dataset. Significant GO terms were projected on the gene ontology tree using the QuickGO browser [30]. To summarize the most relevant functions, terms falling along the same GO tree branch were grouped into a single category identified by a selected representative GO.

### Western blot validation

To validate proteomics data, ten DEPs were selected for analysis by western blot. The same protein lysates used for mass spectrometry were run in a 10% acrylamide gel electrophoresis and transferred to a polyvinylidene difluoride (PVDF) membrane (Millipore). Membranes were blocked with 5% (w/v) non-fat milk diluted in TBST and incubated with primary antibodies (Additional file 1: Table S2) overnight at 4 °C. On the following day, membranes were washed with TBST and incubated with HRP-conjugated goat anti-mouse IgG or goat anti-rabbit IgG (Bio-Rad) secondary antibody (1:3000) for 1 h at room temperature. Chemiluminescent detection was performed using the Clarity™ Western ECL substrate (BioRad) and the Chemidoc™ XRS system (BioRad). The quantification of band intensity was performed using the Image Lab software (BioRad) and normalized to the loading control as appropriate.

### Transcriptomic and proteomic data integration

To integrate the data obtained from the transcriptomic and proteomic analysis, both data sets as well as the UniProt database (HUMAN_9696_idmapping.dat.gz) were loaded into R. Given that this strategy requires column names to be the same, those were renamed in both transcriptomic and proteomic data sets. Transcripts/protein matches were found using the inner_join() function from the dplyr package. First, the UniProt database was joined with the transcriptomics data by the Ensembl ID and the results from this intersection were joined with the proteomics data set by the UniProtKB accession number.

## Results

### CFTR-edited cell lines recapitulate disease-associated mRNA and protein expression phenotypes

The aim of this work was to evaluate the specific effects of prototypical CF-causing mutations, representative of predominant classes, on cellular function. To achieve this purpose, we performed an integrated profiling of the mutation’s impact on the transcriptome and proteome of bronchial epithelial cells. The 16HBE14o- parental cell line was used as the non-diseased reference, in parallel with five gene-edited versions harbouring homozygous CF mutations. These included G542X (class I), F508del and N1303K (class II), and G551D (class III), developed by the Cystic Fibrosis Foundation (CFF) by gene editing of the 16HBE14o- cell line [20]. Additionally, we developed a 16HBE14o- model for the I1234V-CFTR mutation (class V), established with the same gene editing tools used by the CFF.

I1234V is a class V mutation that was previously shown to introduce a cryptic splice site (Additional file 2: Fig. S1A, B) [31]. Using a sgRNA/Cas9-based approach we isolated a clonal cell line containing the I1234V mutation in homozygosity, as confirmed by Sanger sequencing (Additional file 2: Fig. S1C). Considering that this is a splicing mutation, we next evaluated the CFTR alternative splicing patterns in the I1234V-CFTR cells. CFTR cDNA was amplified in nine different PCR reactions using different primer pairs [32]. Sequencing analysis confirmed the aberrant splicing of exon 22 leading to the loss of 18 nts, in line with the previously described impact of the I1234V mutation using a CFTR mini gene (Additional file 2: Fig. S1D).

**Fig. 1:**
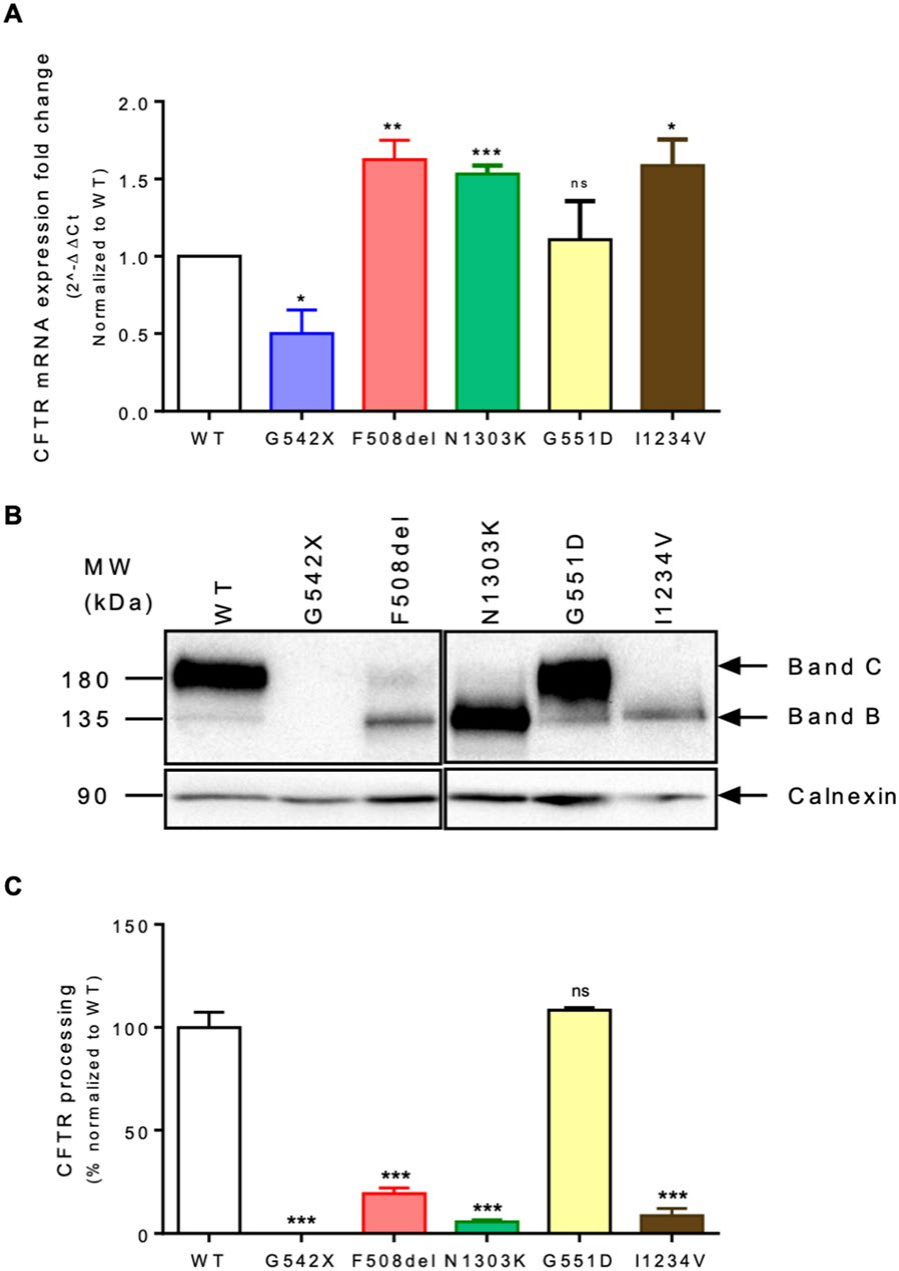
CFTR expression in 16HBE WT-CFTR and CRISPR-engineered cell lines **A** CFTR mRNA expression levels determined by RT-qPCR normalized to GAPDH (housekeeping gene). **B** Western Blot (WB) analysis of CFTR protein expression (UNC596) and Calnexin loading control. **C** CFTR processing (C/C + B) was determined, and results shown normalized to WT-CFTR. Fold-change values are mean ± SEM, relative to WT-CFTR (n = 3 biological replicates). Asterisks indicate * P ≤ 0.05, ** P ≤ 0.01, *** P ≤ 0.001, and ns – not significant

The gene-edited cell lines, as well as the parental one (henceforth referred to by their mutation name or WT, respectively), were assessed for CFTR mRNA and protein expression by RT-qPCR and western-blot, respectively. As shown in Fig. 1, all cell lines display the expected CFTR mRNA and protein expression pattern, recapitulating what was previously described for each mutation and mutation class in other cellular models and in materials from individuals with CF [20, 33]. The G542X cell line shows a reduction in CFTR mRNA levels and no detectable protein. Both F508del and N1303K cell lines have slightly higher levels of CFTR mRNA than the WT but show reduced to absent levels of mature (band C) CFTR protein. The G551D cell line has no differences in CFTR expression and protein processing compared to WT cells. Even though I1234V-CFTR mutation introduces a splice donor site that results in the deletion of 18 nt (six amino acids) in exon 22, as this is an in-frame deletion it has no impact on total CFTR mRNA levels. However, very low levels of mature protein are produced, suggesting that the loss of these six amino acids interferes with CFTR folding and/or stability.

### Transcriptome profiling of CF model cell lines reveals the impact of the mutation class on cellular homeostasis

We used total RNA samples from the parental and gene-edited 16HBE14o- bronchial epithelial cells to characterize the impact of each *CFTR* mutation on the transcriptome. A total of 18 RNA-Seq libraries were generated, corresponding to three biological replicate samples of the six cell lines. These libraries were sequenced to an average depth of 22 M paired-end raw reads (ranging between 20-24 M), of which 88% were retained following quality filtering (see Additional file 3. Data S1). On average, 78% of the quality filtered reads presented unique alignment to the human genome reference, corresponding to a universe of about 13,800 expressed genes (with raw counts > 10).

Exploratory data analysis showed a high level of correlation between biological replicates, underscoring the robustness of the dataset (Additional file 2: Fig. S2A). Interestingly, samples from class II mutation models (F508del and N1303K) were segregated from WT, G542X, G551D, and I1234V samples by both clustering and principal component analysis (PCA) (Additional file 2: Fig. S2). The class III G551D mutation model was found to be most similar to the WT samples, in good agreement with the shared CFTR mRNA and protein expression profiles. Given that all mutations reduce CFTR function, these results suggest that a significant fraction of the changes observed at the level of the transcriptome may be related to responses to aberrant gene expression processes like the accumulation of unfolded or unstable CFTR protein or the activation of RNA degradation pathways, i.e., to the specific mechanisms through which each mutation causes disease (absence of protein, impaired trafficking, defective gating, etc.).

**Fig. 2:**
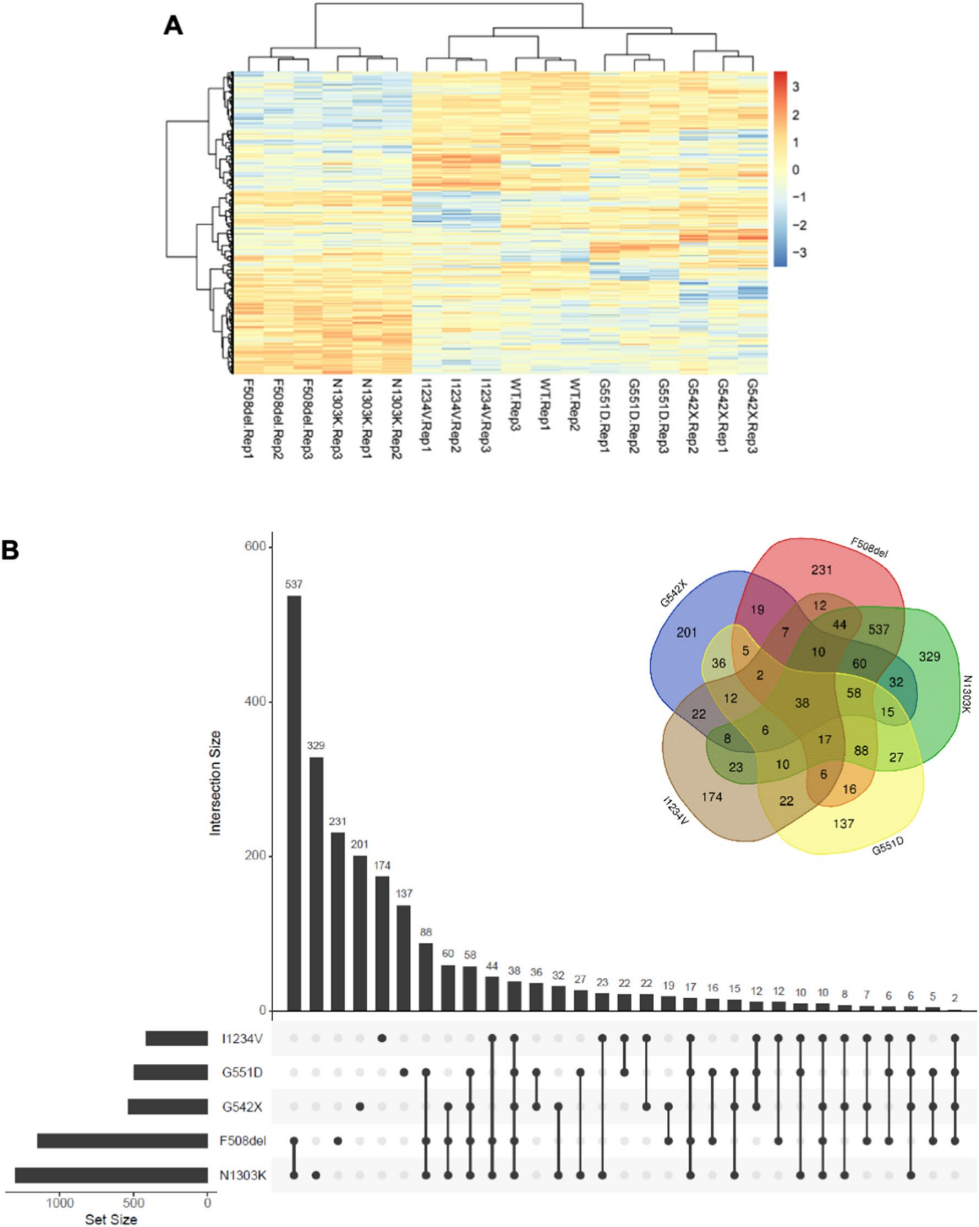
Differentially expressed genes (DEGs) identified in each CFTR-mutant cell line **A** Cluster heatmap analysis of the genes identified as differentially expressed in WT-CFTR and CFTR mutant cell lines. The gradient-coloured barcode at the top right indicates log_2_(CPM). CPM, Counts Per Million reads. **B** Venn diagram and a bar chart showing the number of common and mutation-specific DEGs

To obtain a detailed insight into the specific transcriptome changes induced by each mutation, we performed a differential expression analysis (DEA) for each mutant cell line versus the WT control. The number of differentially expressed genes (DEGs) for each *CFTR* mutation (considering an adjusted p-value < 0.05) ranged from 1304 in the N1303K mutant to 416 in the I1234V mutant (Additional file 4. Data S2). In agreement with what was expected, CFTR was only found to be differentially expressed in the G542X mutant, with a log2 fold change (log2FC) of − 2.5 relative to the WT control (with an adj. p-value of 6.5 × 10^−7^). Figure 2A shows an unsupervised clustering analysis of the expression levels of the DEGs found across all comparisons, again highlighting the highly distinctive profile of samples with class II mutations compared to all other samples.

To address the similarities and differences found across our transcriptome dataset, we performed an overlap analysis of the DEG set for each CF cell line model (Fig. 2B). Once again, the two class II mutations (F508del and N1303K) differentiated from the other classes, not only by having the highest number of DEGs (twice as much as the other *CFTR* mutations) but, particularly, by sharing the highest proportion of DEGs—73% and 65% of the F508del and N1303K DEGs, respectively (Fig. 2B). In contrast, all other mutations shared at most 30% of their DEGs with each other, of which approximately one third (38 DEGs) were shared across all genotypes. This analysis also showed a considerable number of unique DEGs for each genotype, ranging between 137 (for G551D) and 329 (for N1303K) (Fig. 2B).

Taken together, these results suggest that most changes detected at the transcriptome level are more likely to be a consequence of the cell’s attempt to cope with changes in gene expression induced by each type of mutation than to represent a response to the actual loss-of-function of the CFTR channel. These differences may to some extent underlie the phenotypic variability observed in patients and point towards the existence of mutation-specific alterations in cellular pathways that may be of therapeutic interest.

**Fig. 3:**
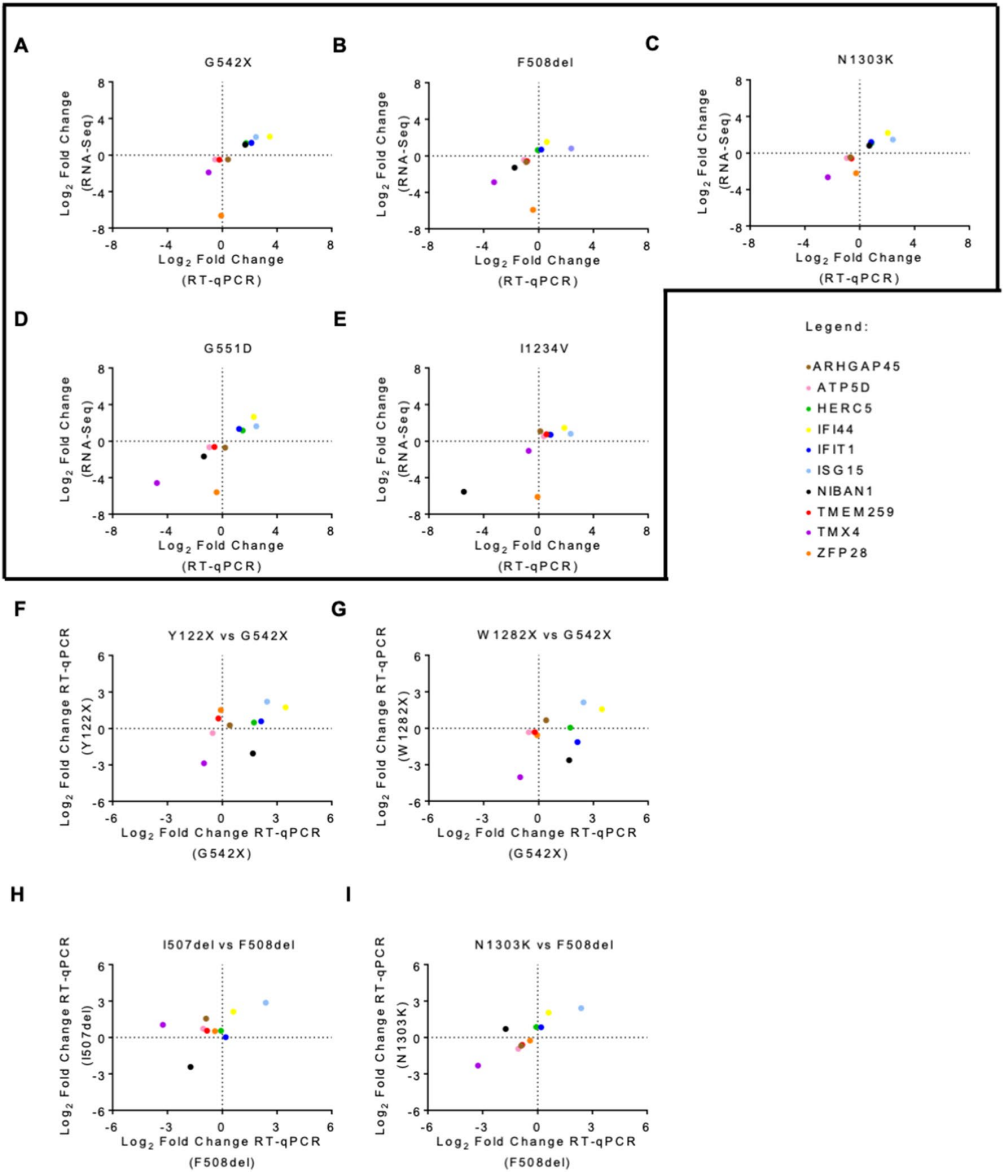
Validation of DEGs via RT-qPCR Ten DEGs common to all mutant cell lines were validated by RT-qPCR: **A** G542X-CFTR, **B** F508del-CFTR, **C** N1303K-CFTR, **D** G551D-CFTR, and **E** I1234V-CFTR. The 2^−ΔΔCT^ method was used for data analysis using GAPDH as a housekeeping gene. The vertical axis represents the gene expression level obtained from RNA-seq, and the horizontal axis represents the gene expression level obtained from RT-qPCR. Correlation analysis of the same DEGs was also performed in similar mutations. Correlation analysis between gene expression obtained from RT-qPCR in each of the class I mutations **F** Y122X-CFTR and **G** W1282X-CFTR vs G542X-CFTR and each of the class II mutations **H** I507del-CFTR and **I** N1303K-CFTR vs F508del-CFTR. Each coloured dot represents a different gene. For a gene to be validated, the corresponding dot must fall either on the bottom left or top right square. All data are presented as mean ± SEM and relative to WT-CFTR (n = 3 biological replicates)

To validate the RNA-Seq analysis we selected ten out of the 38 common DEGs identified across the five different CF genotypes: *ARHGAP45, ATP5D, HERC5, IFI44, IFIT1, ISG15, NIBAN1, TMEM259, TMX4, ZFP28*. These DEGs presented a significant adjusted p-value, but their absolute log2FC covers a wide range of values, from minimal to large variation (0.4 to 6.6) (Table 1). Furthermore, the direction of expression change in this panel of selected genes varies between *CFTR* mutations, thereby providing a broad spectrum of possible results for the assessment of the robustness of our transcriptome profiling. Validation was performed by RT-qPCR using the same samples used for RNA-Seq. In all five genotypes, we could observe a high correlation between RNA-Seq and RT-qPCR, with almost every gene displaying concordant expression values between the two methods (Fig. 3A–E). Overall, RT-qPCR results were highly consistent with the expected, confirming the robustness of our RNA-Seq analysis.

**Table 1:** List of the DEGs and DEPs common to all CFTR mutations

	G542X	F508del	N1303K	G551D	I1234V
Genes	Log_2_ FC				
*ARHGAP45*	− 0.47	− 0.67	− 0.45	− 0.70	1.10
*ATP5D*	− 0.47	-0.43	− 0.53	− 0.64	0.53
*HERC5*	1.30	0.64	1.10	1.18	0.74
*IFI44*	2.90	1.53	2.20	2.67	1.47
*IFIT1*	1.34	0.69	1.19	1.35	0.71
*ISG15*	1.96	0.83	1.46	1.65	0.82
*NIBAN1*	1.15	− 1.29	0.82	− 1.66	− 5.54
*TMEM259*	− 0.50	− 0.57	− 0.59	− 0.62	0.77
*TMX4*	− 1.90	− 2.87	− 2.64	− 4.58	− 1.05
*ZFP28*	− 6.64	− 5.91	− 2.21	− 5.59	− 6.09

Proteins	Log_2_ FC				
BACH	0.19	0.77	0.44	0.39	− 0.24
ERO1A	0.22	0.59	0.46	0.21	0.23
ESYT1	0.26	0.37	0.24	0.14	0.60
FINC	0.57	0.63	0.24	− 0.60	0.74
GARS	− 0.17	− 0.26	− 0.27	− 0.19	− 0.31
RAN	-0.39	− 0.70	− 0.59	− 0.34	− 0.22
RPN1	0.22	0.33	0.16	0.29	0.38
SERPH	− 0.37	− 0.76	− 0.45	− 0.21	0.60
UBP14	0.21	− 0.16	− 0.33	0.40	0.42
VPS35	0.31	0.16	0.15	0.15	0.37

Data are expressed as Log_2_ fold change (FC) in mutant cells versus WT cell line. The orange font represents upregulated genes or proteins while blue font represents downregulated genes or proteins. The values shown here were obtained from RNA-Seq and MS analysis

Given our hypothesis that transcriptome changes are predominantly reflecting the CFTR mutation class, we further evaluated the behaviour of this panel of genes in two other cell lines carrying class I mutations (Y122X and W1282X, obtained from the CFF) [20]. As expected for class I mutations and in agreement with a previous report, Y122X and W1282X have reduced levels of CFTR mRNA and no detectable CFTR protein, thus presenting a similar phenotype to the G542X mutant (Additional file 2: Fig. S3A and B) [20]. A third cell line carrying the class II mutation I507del in the 16HBE14o^−^ background was also used for this analysis (Additional file 2: Fig. S4A). In contrast with the class II cell lines that were used for transcriptome profiling, this cell line presented a reduction of CFTR mRNA levels of ~ 70% when compared to WT-CFTR (Additional file 2: Fig. S4B). At the protein level, western blot analysis confirmed reduced levels of mature CFTR (band C) in I507del-CFTR (Additional file 2: Fig. S4C), as described previously for this mutation in different cell models.

The analysis of this panel of ten genes revealed a strong correlation for class I mutations (approximately 70% and 80% of the tested DEG panel with similar changes in Y122X or W1282X when compared to G542X, respectively) (Fig. 3F, G). Regarding the class II I507del mutant, the correlation with gene expression changes in the F508del was of only ~ 40% of the DEGs being tested (Fig. 3H). This contrasts with the comparison of the RT-qPCR data generated for the validation of the RNA-Seq results for F508del and N1303K class II mutations, which agrees with a very similar transcriptome profile (Fig. 3I). Given that the reduced CFTR mRNA expression profile of the I507del mutant is quite distinct from the very significant up-regulation presented by the F508del and N1303K cell lines (cf. Additional file 2: Fig. S4B with Fig. 1A), this observation is nonetheless consistent with our hypothesis that the major driver of the observed transcriptome is the cell’s attempt to cope with the effects of the mutation on gene expression pathways rather than the impact on CFTR itself.

As there was a great correlation between the two class II mutations, we decided to further validate our RNA-Seq data in primary human nasal epithelial (pHNE) cells homozygous for the F508del and N1303K mutations. In the pHNE-F508del cells, 7 genes showed concordant expression values between the RT-qPCR and RNA-Seq (Additional file 2: Fig. S5A), and, in the pHNE-N1303K cells, 3 genes exhibited concordant expression values between the two techniques (Additional file 2: Fig. S5B). Even though we could validate most of the genes in the pHNE-F508del cells, which agree with our results in the 16HBE-F508del cells, the use of primary cultures introduces interpersonal variability which might explain the results in the pHNE-N1303K cells. Also, the 16HBE cell models used for the RNA-Seq have a different tissue origin than the primary cells used in the validation, as the first come from the bronchia (lower airways) while the second are nasal cells from the upper airways (more accessible), which might also introduce some variability.

**Fig. 4:**
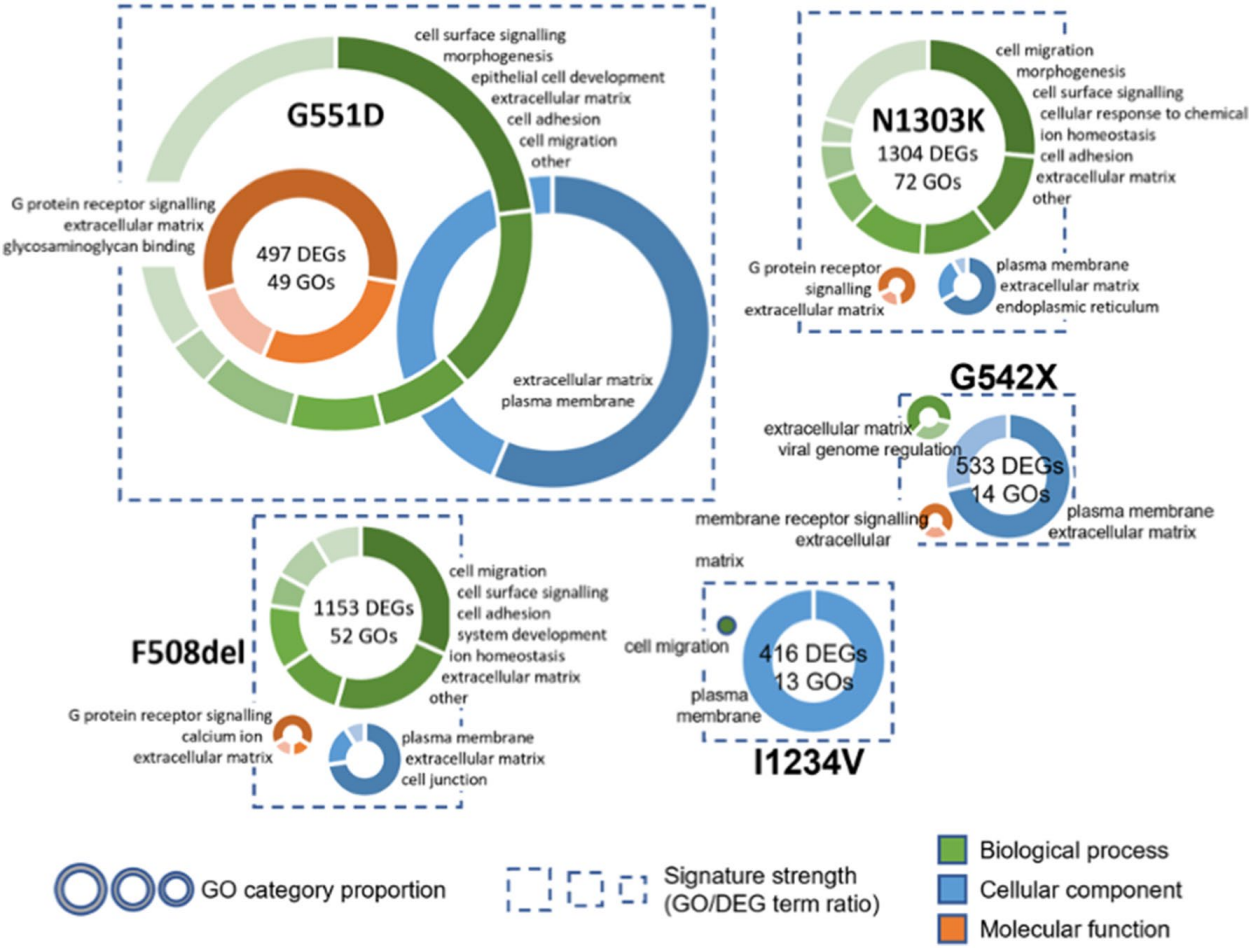
Gene Ontology (GO) enrichment analysis for the DEGs associated with CFTR mutations For each mutation, the size of the dotted squared is proportional to the ratio between the total number of enriched GO terms and the number of DEGs, reflecting the strength of the functional signature in the gene set. Inside each square, the enriched GO terms are summarized by donut charts for each of the three branches of the GO tree—biological process (in green), cellular component (in blue) and molecular function (in orange). The size of each chart is directly proportional to the number of significant GO terms assigned to the corresponding branch (no chart is displayed if no significant terms were found). Donut charts show the number of enriched GO terms grouped by functional categories, according to the selected representative identifier (see main text). Categories are plotted clockwise in the donut chart from highest to lowest number of significant GO terms, with the category identifiers presented by the chart in the same order

To generate insights into the functional impact of each mutation, we performed a gene set enrichment analysis (GSEA) of differentially expressed transcripts regarding the biological process, molecular function, and cellular component Gene Ontology (GO) terms (Additional file 5. Data S3). The GO terms associated with the five DEG subsets were analysed for overrepresentation against the background transcriptome of the corresponding cell line using the hypergeometric test, with the rate of false positives being controlled by using a cut-off for Family Wise Error Rates (FWER) < 0.05 (see methods). To create a visual representation of these results, the GO term tree was inspected to identify enriched GO terms that were located along connected branches and could thus be coalesced into a single representative term (Fig. 4). This approach led to the definition of a range between 2 to 13 candidate cellular processes in each of the three GO categories that are disrupted by the different CF mutations.

Despite the relatively limited overlap of differentially expressed genes across the different cell lines, we find that the functional signatures identified in all of them are very similar, with “cell surface signalling”, “plasma membrane”, and “extracellular matrix” related processes consistently appearing as enriched. It is noteworthy that all these functions are highly related to the expected CFTR-dependent phenotypes. The two class II mutations (F508del and N1303K) again present the most similar profiles, with the biological process GO term “ion homeostasis” appearing as specifically enriched. Given the highly varying number of DEGs and significant GO terms identified in each cell line, we tried to quantify the “strength” of the functional signature within each gene set as the ratio between the number of enriched GOs and DEGs, visualized as the size of the dotted boxes/donut charts in Fig. 4. A high ratio should highlight a very convergent impact of the mutation on a given cellular function, whereas a low ratio will correspond to a more pervasive effect of the mutation across the transcriptome. Interestingly, G551D – the only mutation in which both CFTR mRNA and protein levels are similar to wt—presents the strongest functional signature. It is followed by the two class II mutations which, as mentioned, have very similar behaviour. With more than two times the number of DEGs, the number of enriched GO terms identified in these cell lines is in the same range as the G551D mutation (~ 50). Finally, with a similar number of DEGs to G551D, the transcriptomes of the G542X and I1234V mutations, which are known to act at the level of the mRNA, resulting in the absence or reduced levels of functional CFTR, respectively, display the weakest functional signatures. Indeed, the I1234V splicing mutation presents both the weakest functional signature and the mildest CFTR phenotype. Given the overall similarity of the enriched functional GO terms across all mutant cell lines, and their connection to the expected CFTR loss-of-function phenotypes, the observed differences in functional signature strength are consistent with our hypothesis that the different transcriptome profiles predominantly reflect the cell’s attempt to cope with mutation-specific changes in gene and protein expression. Indeed, if CFTR-dependent processes are the predominant determinant of transcriptome changes a relatively consistent ratio GO/DEG ratio is to be expected. In a similar functional background, variation in the number of DEGs will be a function of sample variability, directly influencing the number of significant GOs due to an increased sensitivity of the hypergeometric function in larger gene sets. This phenomenon is well exemplified by the two class II mutations which, despite a ~ 40% difference in the number of significant GOs, display a very similar signature strength and functional profile. The fact that the functional signature strength in the other three mutations, which have a similar number of DEGs varies ~ fourfold, suggests that the core functional signature is independent of CFTR loss-of-function (LOF). With ~ 500 DEGs, class I (G542X) and class V (I1234V) mutations, which impact very different aspects of mRNA metabolism and protein synthesis, have weak functional signatures, whereas the transcriptome of the class III G551D mutation, with normal CFTR mRNA and protein levels but total loss of CFTR function displays a very focused functional signature towards CFTR-dependent processes, with the total number of significant GOs in the same range of the class II mutations.

### Differentially expressed proteins between wild-type and mutant CFTR confirm a signature that correlates with the mutation class

A global proteome analysis using sequential window acquisition of all theoretical fragment ion spectra (SWATH) mass spectrometry (MS) analysis was performed to complement the transcriptomics data from the same samples. Collectively, 936 proteins were identified across all samples with a 1% false discovery rate (FDR, Additional file 6. data S4). Of the proteins identified, 836 proteins were quantified with a minimum of one peptide with at least three fragments and a maximum of six peptides used in the quantification (Additional file 7. data S5). Principal component analysis was used to assess the differences between mutations as well as to determine possible variations between biological and technical replicates. Consistent with the transcriptomics results, this analysis segregates class II mutations from the other samples while confirming the robustness of the proteomic analysis (Additional file 2: Fig. S6). Interestingly, at the proteome level the samples from class III and V mutations, in which there is (at least some) protein at the plasma membrane, cluster together.

**Fig. 5:**
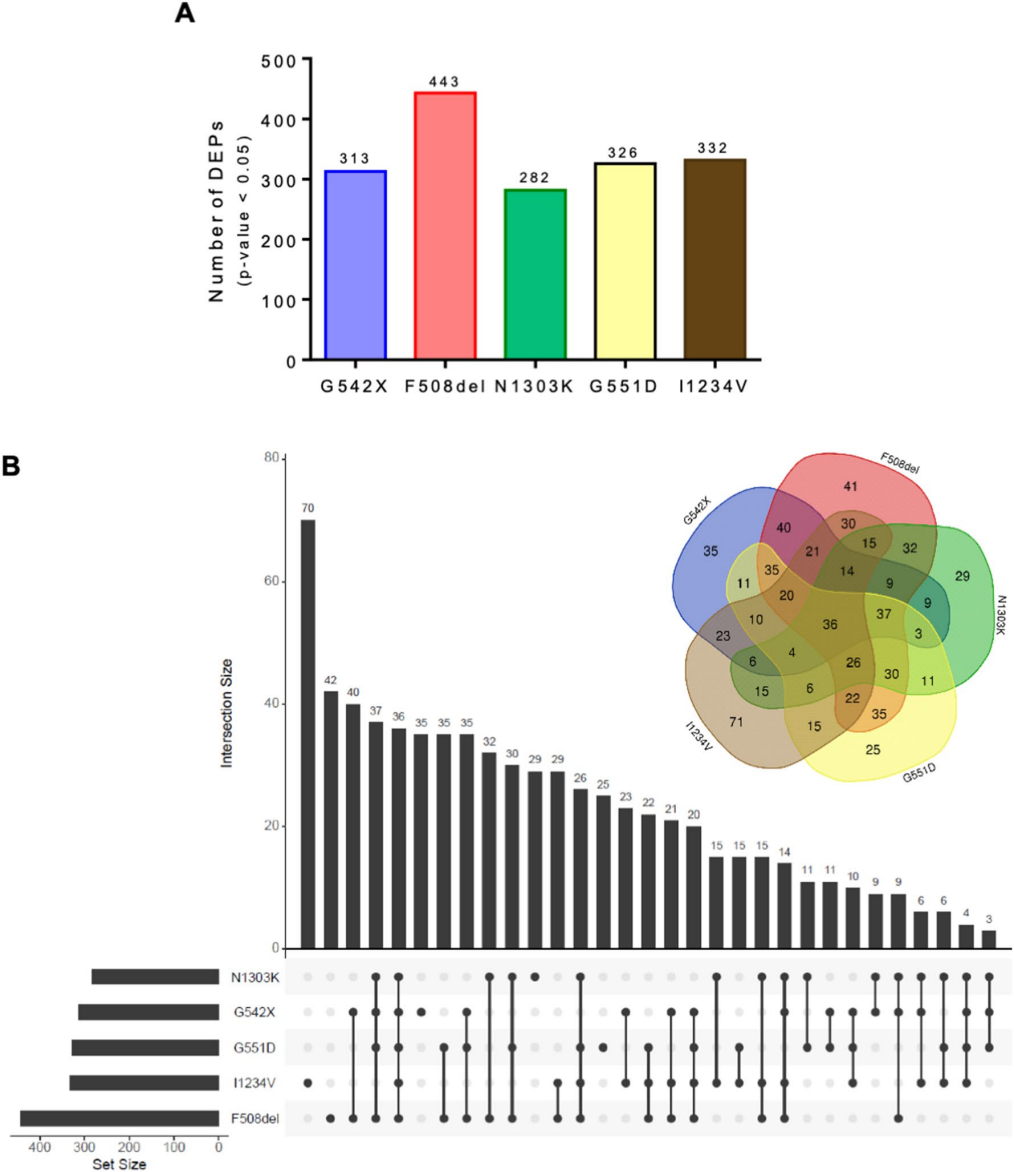
Differentially expressed protein (DEPs) identified in each CFTR-mutant cell line **A** Quantity of DEPs identified for each mutation. **B** Venn diagram and bar chart showing the number of common and mutation-specific DEGs

On average 340 proteins were identified as being differentially expressed between each mutant and the WT cell line (ranging from 282 to 443 DEPs with a p-value < 0.05) (Fig. 5A). Thus, although the total number of proteins detected by the proteomic analysis was two orders of magnitude below the number of detected transcripts, the number of DEPs and DEGs is within a similar range, exception made for the two class II mutations that presented > 1000 DEGs. The results from this analysis with the corresponding expression levels and adjusted p-values can be found in Additional file 7. Data S5. We next looked at the number of unique and shared DEPs between CFTR mutant cell lines. Only 36 of the 443 DEPs in the dataset were common to all mutations. Contrasting with the results of the transcriptome analysis, the proportion of unique DEPs was considerably small, ranging between 8 and 21% of the DEPs, compared to 20–42% of unique DEGs (Fig. 5B). Furthermore, the F508del and N1303K exclusively shared ~ 70% of common DEGs, at the protein level only 32 DEPs were uniquely shared between the two class II mutations, corresponding to ~ 10% of their DEP subset. Notwithstanding, when looking at the global proportion of shared proteins, ~ 70% of N1303K DEPs are in common with F508del, a number that is > 40% higher than the proteins shared with any of the other three mutations.

**Fig. 6:**
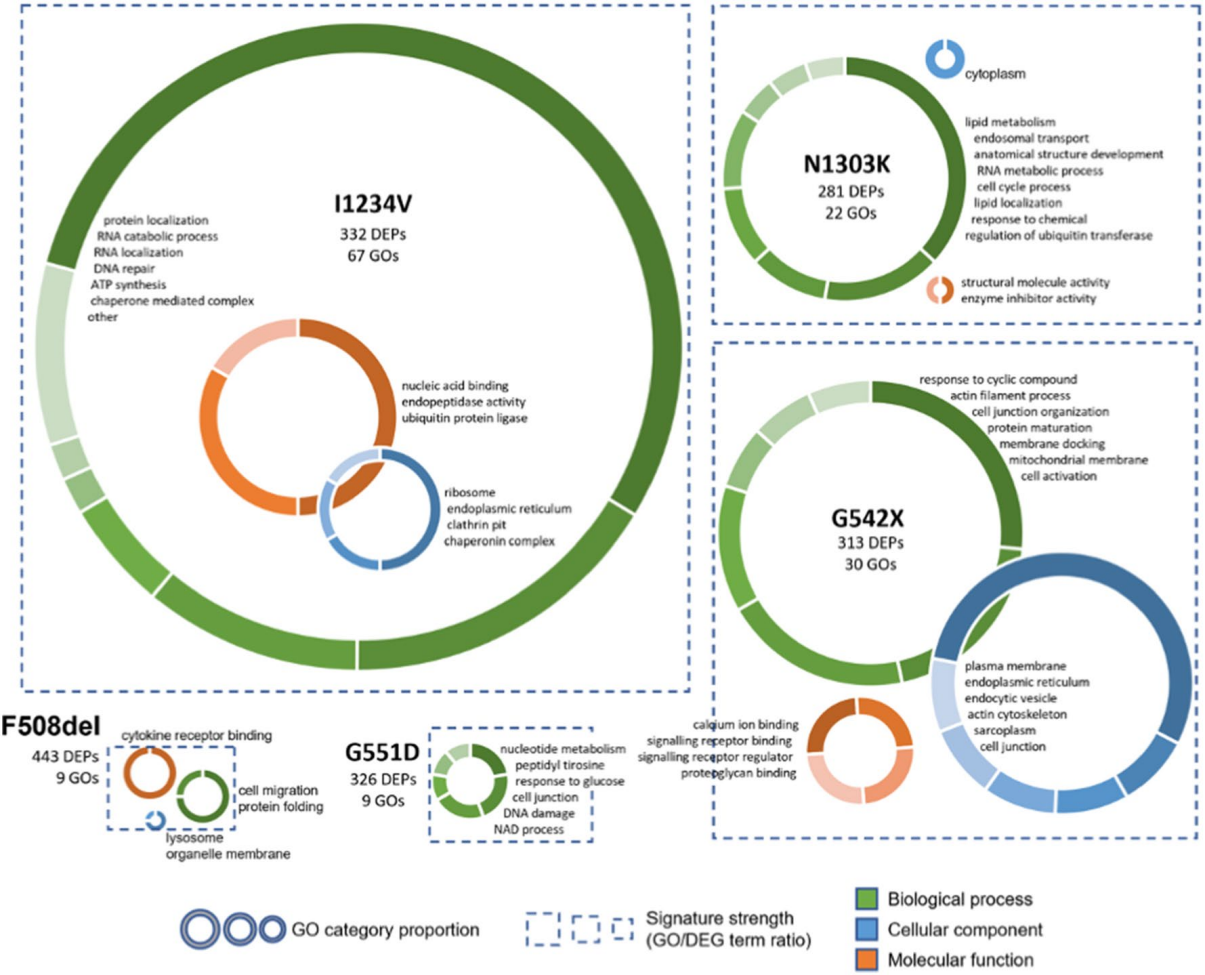
Gene Ontology (GO) enrichment analysis for the DEPs associated with CFTR mutations For each mutation, the size of the dotted squared is proportional to the ratio between the total number of enriched GO terms and the number of DEPs, reflecting the strength of the functional signature in the protein set. Inside each square, the enriched GO terms are summarized by donut charts for each of the three branches of the GO tree—biological process (in green), cellular component (in blue) and molecular function (in orange). The size of each chart is directly proportional to the number of significant GO terms assigned to the corresponding branch (no chart is displayed if no significant terms were found). Donut charts show the number of enriched GO terms grouped by functional categories, according to the selected representative identifier (see main text). Categories are plotted clockwise in the donut chart from the highest to lowest number of significant GO terms, with the category identifiers presented by the chart in the same order

A GO enrichment analysis was also conducted to assess which biological processes, cellular components and molecular functions undergo significant changes at the protein level in the different CFTR mutant cell lines (Fig. 6). Although the number of DEPs was not very different from DEGs, the enrichment of GO terms in comparison to the detected proteome was much less significant than the results obtained for DEGs (Additional file 8. Data S6). This result was not improved by using the transcriptome as background or filtering the DEP sets to remove proteins with lower fold changes (not shown). Notwithstanding, the GO terms that were retained for each CFTR mutation using a significance cut-off of uncorrected p-value < 0.01 had a clear connection with the disease pathophysiology, but unlike the signatures detected in the transcriptome data, these seem to correlate predominantly with the mutation class. Using the strategy described above, we reduced the number of selected GO terms to a maximum of 8 categories for a visual representation depicting the strength of the functional signature (Fig. 6). Interestingly, we find that mutants that displayed strong functional signatures at the transcriptome level show minimal enrichment in the proteome analysis and vice-versa. Indeed, the I1234V mutation, which presents a very limited functional signature at the transcriptome level, has a GO term to DEP ratio over two times higher than all other mutations. Interestingly, given the fact that this is a splicing mutation, the altered biological processes and molecular functions are predominantly linked to RNA/nucleic acid metabolic processes. Furthermore, a high number of DEPs are linked to chaperone/ubiquitin/protein degradation functions, which are in line with the predicted synthesis of a misfolded protein. Protein folding and degradation signatures are also found in the class II mutants F508del and N1303K, in agreement with the reported mutation effect. However, the functional signature of these mutations at the protein level is comparatively weak and quite distinct between the two, in agreement with the limited overlap of DEPs observed. The cell line harbouring the class I G542X mutation has the second strongest functional signature, highlighting effects on signalling regulation, cellular components linked to the ER, endocytic vesicles, and plasma membrane. Contrary to the observation at the transcriptome level, a high number of DEPs identified in this cell line is involved in actin cytoskeleton organization, with cell junction organization also appearing as an enriched term. Finally, the G551D cell line displays a limited functional signature for DEPs, with several terms related to nucleotide metabolism, which could be linked to the inability of this mutant to hydrolyse ATP.

**Fig. 7:**
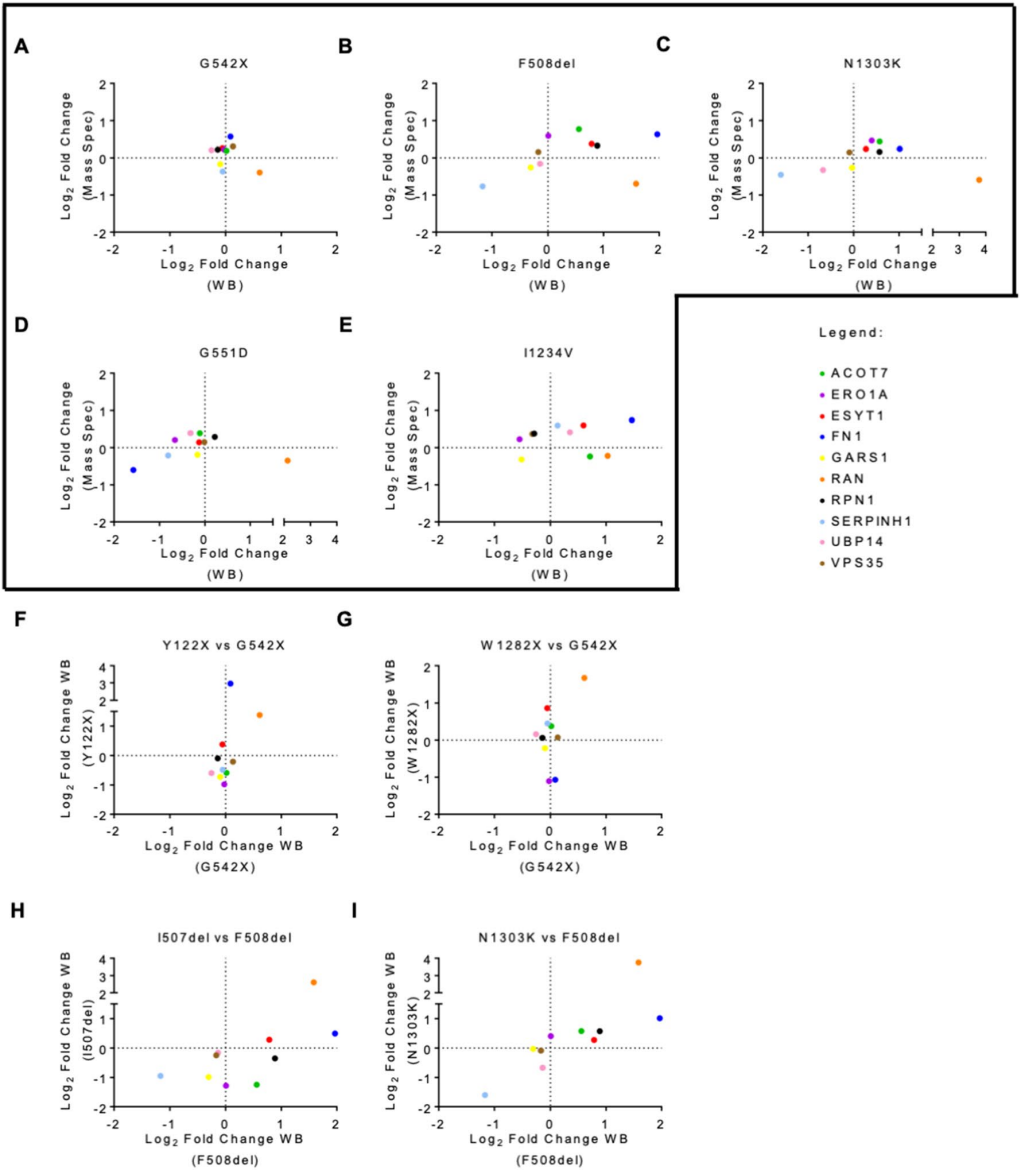
Validation of DEPs via Western blot (WB) Ten DEPs common to all mutant cell lines were validated by WB: **A** G542X-CFTR, **B** F508del-CFTR, **C** N1303K-CFTR, **D** G551D-CFTR, and **E** I1234V-CFTR. The vertical axis represents the protein expression level obtained from mass spectrometry, and the horizontal axis represents the protein expression level obtained from WB. Each coloured dot represents a different protein. Correlation analysis of the same DEPs was also performed in similar mutations. Correlation analysis between protein expression obtained from western blot in each of the class I mutations **F** Y122X-CFTR and **G** W1282X-CFTR vs G542X-CFTR and each of the class II mutations **H** I507del-CFTR and **I** N1303K-CFTR vs F508del-CFTR. For a protein to be validated, the corresponding dot must fall either on the bottom left or top right square. All data are presented as mean ± SEM and relative to WT-CFTR (n = 3 biological replicates)

As before, we selected ten common DEPs—GlyRS, FN, ESYT1, USP14, ERO1A, ACOT7, VPS35, RPN1, HSP47, and RAN—with different expression patterns among the *CFTR* mutations to validate the mass spectrometry results by western-blot (Table 1). The overall validation rate by western blot for the expression change observed in mass spectrometry was about 50%, supporting the reliability of the proteomics data obtained (Fig. 7A–E). The correlation between mass spectrometry and western blot data is lower than the observed between RT-qPCR and RNA-seq data, which may be partly explained by the fact that western blot is less robust as a quantitative approach. Adding to this, the 10 proteins were chosen out of the limited set of 32 shared DEPs, presenting relatively subtle—and thus hard-to-detect—fold changes.

We also evaluated the behaviour of this panel of 10 proteins in the other available class I and class II cell lines. Compared to the quantification of mRNA levels for the panel of selected DEGs, this analysis showed a rather limited correlation of protein expression levels between class I mutations (Fig. 7F, G). This may be because the average absolute fold change for this group of proteins in the G542X cell line is only 20% (Table 1). Strikingly, the I507del cell line shows a better correlation with the F508del mutant at the protein level than observed for transcripts, with ~ 70% of the DEPs presenting similar expression patterns (Fig. 7H). Furthermore, the expression of this set of DEPs was very highly correlated between the N1303K and F508del class II mutants (Fig. 7I). This strong similarity occurs despite the differences observed in the GSEA analysis and of the small number of class II specific DEPs. Taken together, these observations further support the proposal that the mutation class is a key determinant of broad proteome changes.

The expression of these 10 common proteins was also evaluated in the primary cells homozygous for the F508del and N1303K mutations. In both genotypes, we identified 2 proteins for pHNE-F508del and 4 proteins for pHNE-N1303K with good correlation between mass spectrometry and western blot (Additional file 2: Fig. S5C and D). As previously mentioned, these results might be explained by different factors: (i) the robustness of the western blot technique, (ii) subtle fold changes in the proteins chosen, (iii) interindividual variability present in primary cultures, and (iv) different tissue origin between pHNE and 16HBE.

### Integration of transcriptomics and proteomics data

**Fig. 8:**
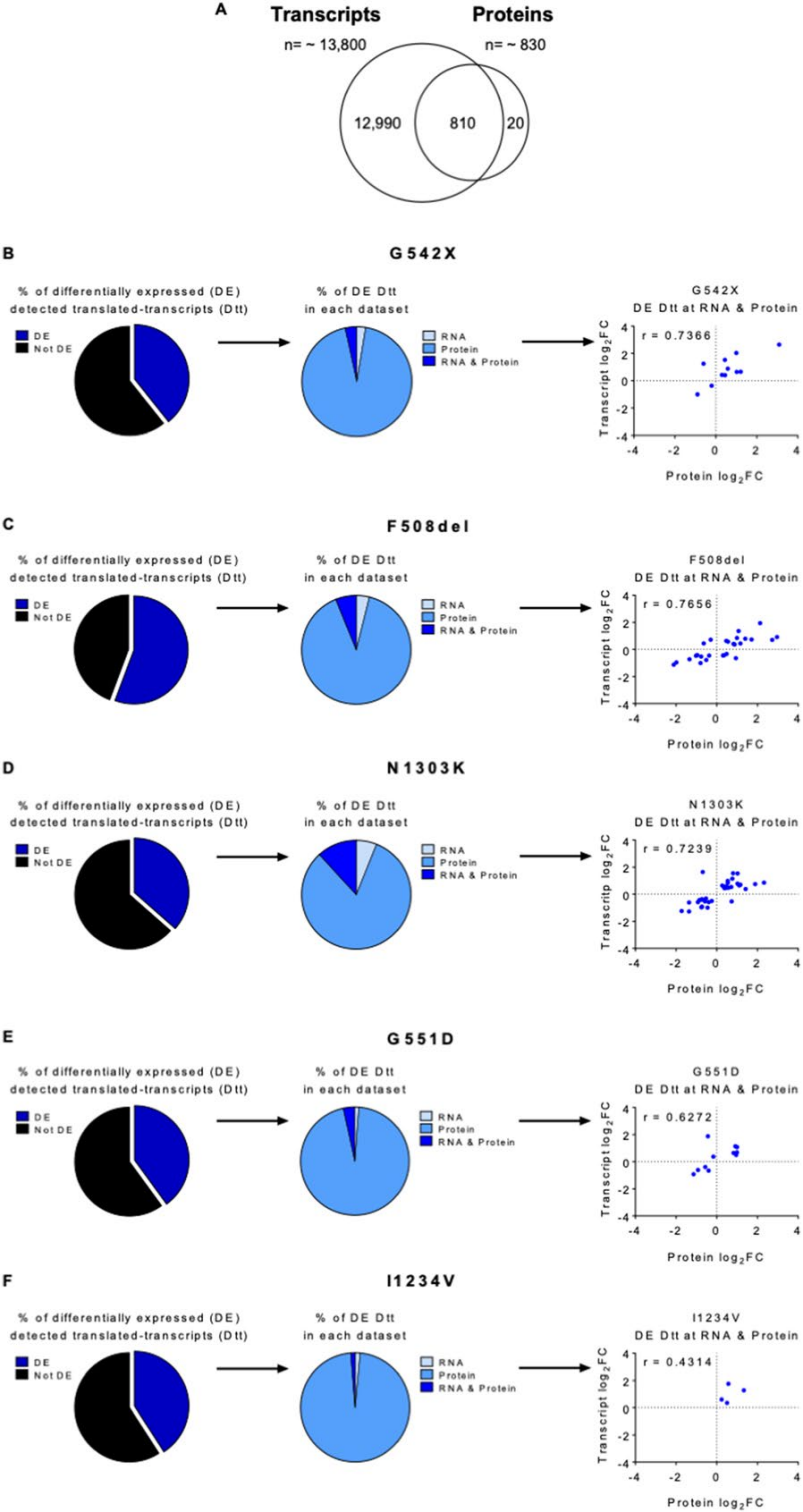
Integration and correlation of transcriptomics and proteomics data sets **A** Integration of transcriptomic and proteomic data showed that ~ 810 proteins in the proteomics dataset have a match in the transcriptomics dataset. Results are represented in a Venn diagram. **B**–**F** Left panels: percentage of differentially expressed (DE) and not DE genes/proteins for each CFTR mutant cell line; Middle panels: Among the DE genes/proteins some were only differentially expressed in the transcriptomics dataset (RNA), others in the proteomics dataset (Protein), and others in both datasets (RNA & Protein); Right panels: correlation between RNA and protein expression levels of the gene/proteins significant in both datasets (RNA & Protein, so-called gene/protein pairs) in the different mutant CFTR cell lines

Given the distinct results described above, we decided to integrate and perform a joint analysis of the two types of omics data to better understand the correlation between the transcriptome and proteome in each of the CFTR-mutant cell lines. The intersection of the total number of transcripts and proteins identified showed that about 810 (97.3%) of the total 836 proteins quantified by mass spectrometry across all samples were encoded by transcripts identified in the RNA-seq data, hereinafter termed “detected translated-transcripts” (Dtt) (Fig. 8A, Additional file 9. Data S7). To understand how well the absolute abundance of these proteins correlates with that of the matching transcripts in each *CFTR* mutation, we performed an across-gene correlation analysis [18]. This analysis revealed a consistent correlation coefficient of approximately 0.4, meaning that 60% of the variability in protein levels can be explained by the variability in transcript levels (Additional file 2: Fig. S7A-F). This result agrees with previous studies of human tissue, including the lung [18, 34].

Most of the Dtt identified were not found to be differentially expressed (DE) as a transcript or protein in any mutant CFTR cell lines except for F508del, where the fraction of DE Dtt is ~ 60% (Fig. 7B–F left panel). Of note, differential expression was predominantly identified at the protein level only, suggesting that most proteins affected by *CFTR* mutations are responding to post-transcriptional and most likely, post-translational regulatory processes (Fig. 7B–F middle panel). For Dtt with altered mRNA levels, DE at the protein level was also observed in most cases. In total, we found 11 such cases in G542X and G551D, in addition to 27, 35, and 4 “concordant” Dtt in F508del, N1303K and I1234V, respectively (Table 2). A strong correlation between the mRNA and the protein levels was observed, with only a few cases with discordant expression levels (r = 0.43–0.77, Fig. 7B–F right panel). Interestingly, class II mutations F508del-CFTR and N1303K-CFTR were the ones showing the highest number of Dtt that seem to be transcriptionally regulated. Dtt differentially expressed at both mRNA and protein levels are listed in Table 2, highlighting in bold those that are specific to a single mutation. No Dtt was common to all mutations. Regarding the DE Dtt that are shared by two or more cell lines, the biggest overlap occurs between F508del and N1303K – with 15 common Dtt, 13 of which are exclusive of this pair. This observation further supports the hypothesis that the global changes observed correlate with the mutation class.

**Table 2:** List of the differentially expressed detected translated-transcripts (Dtt) at the mRNA and protein levels

G542X	F508del	N1303K	G551D	I1234V
ALPK3	**ACTC**	AHNK	**CADH1**	CAN1
CLD6	**AFG32**	BGH3	CAN1	K1C19
CNDP2	AHNK	CAN1	**COF1**	**K2C5**
EMAL3	BGH3	CCAR2	**INO1**	**NALP2**
GANAB	**CADH3**	CLD6	K1C14	
HNRL2	CCAR2	CPT1A	**K2C80**	
K1C14	CLD6	**ERO1A**	**LRBA**	
K1C17	CPT1A	K1C17	OTUB1	
MYL6	**CTL2**	K1C19	**PLST**	
UBXN1	**EHD1**	**K1C9**	**TBL3**	
ZCCHV	**GSDME**	**K2C6A**	ZCCHV	
	**GSTO1**	ML12A		
	K1C17	MRE11		
	**KI13A**	**NB5R1**		
	**KRT86**	NUMA1		
	ML12A	OTUB1		
	MRE11	**PAI2**		
	NUMA1	**PDIA4**		
	OTUB1	**PICAL**		
	**PKP4**	**PNCB**		
	PNPH	PNPH		
	TACD2	**PURA2**		
	TBB6	**RAB1B**		
	TENA	**SFXN1**		
	**TOP2A**	**SQOR**		
	VDAC3	**SSBP**		
	YAP1	TACD2		
		**TBA4A**		
		TBB6		
		**TELO2**		
		TENA		
		**VATB2**		
		VDAC3		
		YAP1		
		ZCCHV		

Mutation-specific Dtt are represented in bold. Dtt shared between F508del and N1303K are underlined

Overall, the integration of transcriptome and proteome data reveals a degree of correlation between transcript and protein levels in agreement with previous reports from the literature. The limited sensitivity of the mass-spectrometry analysis compared to RNA-seq creates a “blind spot” for a very large number of expressed genes whose changes cannot be assessed at the proteome level. Our results suggest that a significant part of the physiological adaptation of the cell to the presence of CFTR mutations happens through the regulation of protein stability or translation efficiency, without any specific impact on the transcriptome level, and justifying the distinct functional signatures observed in the two types of omics profiles.

## Discussion

CF patients present a wide variability in phenotypic features, even those with identical mutations in the *CFTR* gene. While some aspects of the disease can be directly associated with the *CFTR* genotype, the severity of the lung phenotype cannot be predicted solely by the LOF of this gene, implicating the contribution of other genes and non-autonomous cell responses. The phenotypic heterogeneity in CF can thus be attributed to genetic modifiers that remain largely unidentified. Significant technological advances in the fields of transcriptomics and proteomics currently allow us to perform the systematic characterization of individual biological samples within a reasonable time frame. These techniques hold great promise for the identification of genetic modifiers, with their encoded proteins becoming candidate targets for therapeutic intervention [35, 36].

There are a few transcriptomics studies performed in airway tissue samples from CF patients or models, but the vast majority refer to the F508del-CFTR mutation and still use microarray technology [9, 37–44]. However, RNA-Seq technology, used in our study, has several advantages over microarrays including full sequencing of the whole transcriptome at higher sensitivities, including a more robust detection of differentially expressed genes, and of genes with low expression levels [45].

Since proteins are the ultimate effectors of most biological processes, proteomics studies can provide insights into the mechanisms by which CFTR mutations and other genetic modifiers lead to the disease phenotype [46]. Furthermore, they can also identify differentially expressed proteins that can be validated as novel therapeutic targets [35]. There are only a few studies addressing the global protein expression profile in CF airway tissue, the majority of which are also on F508del-CFTR [16, 47–49]. Our study is the first to combine whole transcriptome and proteome analysis of human bronchial epithelial cells homozygous for different CFTR mutations: G542X, F508del, N1303K, G551D, and I1234V. To support the study of the cellular transcriptome and proteome within a consistent genetic background, where the only difference between cell lines is the targeted CFTR variant, we used isogenic cell lines developed through CRISPR/Cas9 mutagenesis [20]. Besides recapitulating all previous knowledge for these mutations (Fig. 1), these cell lines only express endogenous CFTR, thus eliminating the potential disadvantages that arise from the over-expression of a cDNA under a viral promoter.

In our study, we started by investigating the impact that the different CFTR mutants have on the cellular transcriptome compared to WT-CFTR. The expression of ~ 13,800 genes was detected in our RNA-seq dataset for all genotypes analysed. Sample clustering based on gene expression assigned the CFTR mutations to two main groups, clearly differentiating class II mutations from WT cells and the other three mutations/classes (class I, III, and V). Statistical analysis of each CFTR mutant vs WT control allowed us to identify a large set of DEGs, with the two class II cell lines (F508del and N1303K) standing out with 2–3 times more DEGs than the other cell lines (Fig. 2 and Additional file 2: Fig. S2).

Comparison between the different DEGs identified for each CFTR mutation allowed us to identify mutation-specific genes that help define a gene signature which may be relevant for diagnosis and/or therapy. Common to all the CFTR mutations analysed, there were 38 DEGs – 9 of which were successfully validated by RT-qPCR (among 10 tested) (Fig. 3). Two of these genes—ISG15 and HERC5—are up-regulated in all CFTR mutations. The first encodes a protein belonging to the family of ubiquitin-like modifiers, which can modify proteins at the post-translational level. ISG15 protein forms conjugates with proteins—in a process known as ISGylation—through the sequential action of three enzymes (E1, E2, and E3), being HERC5 the major E3 enzyme for human ISG15 [50]. Even though this protein has been mainly studied for its function as an antiviral molecule, it is involved in many other cellular functions including, tagging of potentially pathogenic proteins and clearance of protein aggregates [51, 52]. Although it has never been demonstrated that CFTR undergoes ISGylation, HERC5 has been previously shown to interact with F508del-CFTR in the CF bronchial epithelial (CFBE14o-) cell line [53]. The consistent up-regulation of ISG15 and HERC5 transcripts in all CFTR cell lines suggests the associated molecular pathway may be of relevance in the context of CF. The proteins encoded by these two genes were not found among the set of 836 proteins identified by our quantitative mass spectrometry–based proteomics analysis in all CFTR genotypes. We believe the large discrepancy between the number of identified genes and proteins is explained by the lower sensitivity of the proteomics methods, predominantly affecting the identification of low-abundance proteins [54]. Thus, the lack of overlap between DEGs and DEPs cannot be taken to imply that the detected changes in mRNA levels do not have a corresponding effect at the level of protein and as such, that the former are not functionally relevant. Indeed, our results suggest that combining the two approaches can provide unique insights into the cellular pathways affected by a mutation.

From the 36 DEPs common to all mutations, we chose 10 for validation through western blot. Despite the technical limitations, which include a lack of good commercially available antibodies, three of these DEPs were consistently validated in all the cell lines – GARS, FINC, and SERPH (Fig. 7). GARS is a glycyl-tRNA synthetase whose function is to covalently attach glycine to its cognate tRNA, a process essential for protein translation [55]. This protein has been previously identified in the proteome of HBE cells expressing WT-CFTR, and in the WT-CFTR interactome reported in two independent studies [14, 56]. FINC or fibronectin plays an important role in cell adhesion, migration, growth, and differentiation [57]. A previous study identifies this protein as a mediator of CFTR interaction with EPAC, NHERF1, EZRIN, and SYK, previously known to be involved in the CFTR plasma membrane stabilization, suggesting the indirect role of fibronectin in this process [58]. SERPH or serpin H1 is a collagen-specific molecular chaperone essential to correct the folding of procollagen in the endoplasmic reticulum. It has been demonstrated that SERPH is directly connected with HSF1, a heat shock transcriptional factor that plays a central role in the activation of the heat shock response, which has been implicated in the quality control for misfolded CFTR [48]. Thus, as for the transcriptome analysis, the common changes identified across the five distinct genotypes can be linked to molecular pathways involving CFTR. Also as before, a close similarity between cells expressing the two class II mutations—F508del and N1303K was identified (Additional file 2: Fig. S6). These results support the proposal that the specific class of the mutation imposes a strong gene expression signature that is independent of the CFTR LOF phenotype.

We used gene ontology enrichment analysis to interpret the global changes in gene expression caused by the different CFTR mutations in the bronchial epithelial cells at the transcriptome and proteome levels (Figs. 4, 6). Interestingly, although the number of DEGs common to all mutations is low, the enrichment analysis highlighted a response of the cellular transcriptome with significant overlap between the different mutations. Among the enriched GO terms for the molecular function, we consistently find G protein-coupled receptor signalling, which includes small GTPase signalling pathways, Wnt signalling pathway, and ERK1 and ERK2 cascades. These pathways have been identified as altered in CF in many transcriptomic studies on airway epithelial cell lines [9, 37, 39, 42, 44]. Having noticed a disconnection between the number of DEGs and the number of significantly enriched GO terms, we defined the strength of the functional signature as the ratio between the number of GO terms identified and the number of DEGs. When a higher-than-expected number of gene products with altered expression are annotated to the same function (i.e. GO term), we take this to represent that the function is disrupted in the experimental condition being assessed. Given the nature of the statistical test used in the GO enrichment analysis, larger gene sets will lead to more significant p values when the same proportion of genes is annotated to a specific GO term. Thus, a higher number of GO terms is expected to pass the cut-off criteria if the degree of detected functional disruption is similar to what is found in a smaller gene set. The fact that we do not observe this in the very large DE gene sets for class II mutations versus, for example, the G551D mutation suggests that the additional genes with disrupted expression, albeit common between the two mutations, are not linked to the same biological processes. We propose this reflects the plethora of mutation-specific side-effects targeting different biological mechanisms, thus not presenting a strong functional signature. It is quite striking that the class I and V mutations, which are expected to impact multiple and distinct aspects of RNA metabolism in addition to CFTR LOF, have the weakest functional signatures; and that the G551D mutation, which leads to the presence of non-functional CFTR protein, in normal levels and at its normal location, with correct modifications and folding, displays the most coherent functional signature.

The gene ontology enrichment analysis performed for DEPs (Fig. 6) identified signatures which tend to be very mutation-specific, suggesting that the perturbation of cellular homeostasis caused by each mutation triggers a response at the translation and protein stability level that is somehow related to the mechanism through which a specific mutation leads to CF and reflects the cell’s attempt to cope with the resulting issues. Interestingly, even for the two class II mutations—that are clustered together by the PCA analysis—the enriched terms are diverse, suggesting that although leading to a similar cellular phenotype (defective trafficking), the pathways involved in protein retention are different—which agrees with the very limited response of N1303K-CFTR to the highly effective modulators already approved for F508del-CFTR [59, 60]. These observations are very much in line with the results obtained by the integrated analysis of the transcriptome and proteome datasets.

While gene and protein studies separately can give us indications of the biological processes involved in disease, the integration of both datasets helps us to understand the flow of information from genotype to phenotype and can be valuable to pinpoint potential drug targets [18]. From the total 836 proteins identified in our proteomics approach, ~ 810 have matched transcripts found in the transcriptomics dataset (Fig. 7). This corresponds to 97% of the total protein set and 6% of the total transcripts, consistent with previous studies integrating transcriptomic and proteomic data from human lungs [61]. We designated this gene subset as detected translated-transcripts (Dtt), of which between 40–60% were found to be differentially expressed, depending on the different CFTR cell lines. Interestingly, the vast majority of these were differentially expressed only in the protein dataset. These discrepancies between protein and RNA abundance could be possibly attributed to distinct post-transcriptional and post-translational regulation. Interestingly, the Dtt differentially expressed in both datasets showed a very high correlation between transcript and protein fold changes (Pearson correlation coefficient of ~ 0.70, except for the I1234V cell line, possibly due to the low number of identified Dtts). This suggests that this specific gene subset may be regulated at the transcriptional level.

The lack of common DE Dtts between all mutations again suggests that a core part of the molecular response that can be detected is not linked to CFTR LOF. As before, the two class II mutants share approximately half of their Dtts (16 out of 28–35). To obtain further functional insights on this, the GO annotations for each of these Dtts were manually retrieved and inspected. Dtts identified for the F508del and N1303K are predominantly implicated in processes such as proteolysis, protein folding, protein stabilization, ER to Golgi and Golgi to plasma membrane transport. This agrees with the nature of class II CFTR mutations, which cause protein misfolding and protein retention in the endoplasmic reticulum, leading to premature degradation of CFTR and preventing its trafficking to the cell membrane. The fact that the Dtt involved in these processes are not exactly the same suggests that the two mutant CFTR proteins undergo different degradation pathways. Adding to this, when comparing the differentially expressed genes and proteins common to the two class II mutations (F508del and N1303K) we found two genes (PDZD4 and FLI1) and two proteins (ECHA and TBC14) with opposite expression levels. These opposite expression levels may be indicative of specific mechanisms that may explain the difference observed in the response to therapeutic approaches for the correction of these mutations. Interestingly, the 16 Dtt common to F508del and N1303K that are common (and specific) to the pair suggests an epithelial to mesenchymal transition (EMT) signature associated with these class II mutations. EMT is a developmental process in which polarized epithelial cells are reprogrammed to assume a mesenchymal cell phenotype that includes enhanced migratory capacity and has previously been associated with CF [62, 63].

In summary, the present study provides a global picture of the genes and proteins that are differentially expressed in human bronchial cells as a result of prototypical CFTR mutations, being the first to integrate the transcriptome and proteome of cells carrying not only F508del but also other CFTR mutations. Although the functional signatures identified in the transcriptomics analysis may be associated with the lack of functional CFTR, globally our results reveal a surprising scenario—that the core changes in cell function that are detected at the RNA and protein levels are characteristic of the type of mutation and not so much of the associated gene LOF or “diseased” phenotype. These observations suggest that the approaches used can capture the subtilities of the phenotypic diversity, by detecting signatures that highlight the direct impact of the disease-causing mutation on gene expression processes rather than easily identifying the critical molecular hubs affected by the LOF of the disease-associated gene, which would be relevant as novel biomarkers or therapeutic targets.

Our work thus brings forth a word of caution towards the use of omics approaches to assess disease states, namely when a single, predominant mutation is systematically studied, as is the case of F508del in cystic fibrosis. It further highlights the critical aspect of performing the studies in a biologically relevant model. Indeed, despite our effort to address the impact of CFTR LOF on homozygous, endogenous CFTR mutants in the context of the same bronchial epithelial cell line, this remains an artificial system, lacking the tissue polarisation, structural and cellular interactions present in the context of a human lung. It is possible that in the right physiological context, the molecular changes that connect directly to the disease phenotype will become more apparent. Alternatively, the signatures may be even more confounded by the increased complexity of the system. Taken together, our work provides important new insights that extend beyond the molecular pathology of CF and CFTR LOF to the global quest for the identification of molecular targets for genetic disorders using transcriptomics and proteomics approaches.

## Supplementary Information

**Additional file 1: Table S1.** RT-qPCR primers sequences used for transcriptomics data validation.. **Table S2.** Primary antibodies used for proteomics data validation.

**Additional file 2: Figure S1.** Characterization of the 16HBE I1234V-CFTR cell line A) The A > G alteration at position 3700 showed in red results in the creation of a cryptic donor splice site (GU). B) Scheme of splicing possibilities that occur in the presence of A (in green, WT) or a G (in red, I1234V) at position 3700 corresponding to normal or alternative splicing, respectively. C) Sequence of the WT cells (top panel) and the 16HBE I1234V-CFTR cell line (bottom panel). D) Sequence of the CFTR cDNA in the WT cells (top panel) and I1234V-CFTR cells (bottom panel) showing the aberrant transcript lacking 18-nt. **Figure S2.** Quality of the transcriptomics data A) Correlation of RNA-Seq data between each CFTR mutation replicate. B) Principal component analysis of the normalized RNA-Seq data for each genotype. **Figure S3.** Characterization of the CFF 16HBEge CFTR Y122X and CFF 16HBEge CFTR W1282X generated by the CFF Labs A) CFTR mRNA abundance normalized to GAPDH (house-keeping gene). Fold-change values are mean ± SEM relative to WT (n = 3 biological replicates). Vs. WT: **P ≤ 0.01. B) Western blot analysis of CFTR (UNC596) and Tubulin loading control for WT-CFTR, Y122X-CFTR, and W1282X-CFTR. **Figure S4.** Characterization of the 16HBE I507del-CFTR cell line A) Sequence of the 16HBE WT-CFTR (top panel) and the I507del-CFTR (bottom panel) cell lines confirming the genotype. B) CFTR mRNA abundance normalized to GAPDH (housekeeping gene). Fold-change values are mean ± SEM relative to WT (n = 3 biological replicates). Vs. WT: *P ≤ 0.05. C) Western blot analysis of CFTR (UNC596) and Tubulin loading control for WT-CFTR and I507del-CFTR cell lines. **Figure S5**. Validation of DEGs and DEPs in primary human nasal epithelial (pHNE) cells. DEGs common to all mutant cell lines were validated by RT-qPCR in A) F508del-CFTR and B) N1303K-CFTR cells. The 2-ΔΔCT method was used for data analysis using GAPDH as a housekeeping gene. DEPs common to all mutant cell lines were validated by WB in C) F508del-CFTR and D) N1303K-CFTR cells. The vertical axis represents the gene or protein expression level obtained from RNA-seq or mass spectrometry, respectively. The horizontal axis represents the gene or protein expression level obtained from RT-qPCR or WB, respectively. Each coloured dot represents a different gene or protein. For a gene or protein to be validated, the corresponding dot must fall either on the bottom left or top right square. All data are presented as mean ± SEM and relative to WT-CFTR (n = 3 biological replicates). **Figure S6.** Quality of the proteomics data Principal component analysis (PCA) of the normalized proteomics data for each genotype. **Figure S7.** Correlations between mRNA and protein levels Across-gene correlation analysis comparing absolute mRNA abundance (expressed in fragments per kilobase of transcript per million mapped reads (FPKM)) to protein abundance (expressed as sequential window acquisition of all theoretical mass spectra (SWATH-MS) intensity) in the different cell lines. A) WT-CFTR, B) G542X-CFTR, C) F508del-CFTR, D) N1303K-CFTR, E) G551D-CFTR, and F) I1234V-CFTR.

**Additional file 3: Data S1. **List of RNA-seq samples and the percentage and number of mapped reads.

**Additional file 4: Data: S2.** List of differentially expressed genes (DEGs) in the different 16HBE mutant cell lines (p-value < 0.05).

**Additional file 5: Data 3.** List of significantly enriched GO terms found in the DEG dataset for each 16HBE mutant cell line.

**Additional file 6: Data S4.** List of proteins detected in the different 16HBE cell lines.

**Additional file 7: Data S5.** List of differentially expressed proteins (DEPs) in the different 16HBE mutant cell lines (p-value < 0.05).

**Additional file 8: Data S6.** List of significantly enriched GO terms found in the DEP dataset for each 16HBE mutant cell line.

**Additional file 9: Data S7.** List of genes in the transcriptomic dataset with correspondence in the proteomic data set (gene/protein pairs) in each 16HBE mutant cell line.

## Data Availability

The raw RNA-Seq dataset is available through the European Nucleotide Archive under the study accession number PRJEB46396.
