# Supplementary material for: Mutation-class dependent signatures outweigh disease-associated processes in cystic fibrosis cells
Source: Cell Biosci. 2023 Feb 9;13:26. doi: 10.1186/s13578-023-00975-y (PMC9912517; doi:10.1186/s13578-023-00975-y)
Supplement: Supplementary file 1 — Additional file 1: Table S1. RT-qPCR primers sequences used for transcriptomics data validation.. Table S2. Primary antibodies used for proteomics data validation. [file 13578_2023_975_MOESM1_ESM.pdf]

Additional file Tables

Table S1. RT-qPCR primers sequences used for transcriptomics data validation.

Gene	Forward primer (5' - 3')	Reverse primer (5' - 3')	PCR length (bp)
ARHGAP45	GCCCCATCATGTTCTCCAGG	GGGCAGCTCCCCCGA	107
ATP5D	TGTTGGACCTGGGGGCAG	GGTACGCACCGCCTACTCC	149
HERC5	AGTAGACGAACTCTTGCACCG	TTTTCTGAAGCGTCCACAGTCA	87
IFI44	TGGGAGCTGGACCCTGTAAA	TTCCTCCCTTAGATTCCCTATTTGC	120
IFIT1	ATTTACAGCAACCATGAGTACAAA	TCCCACACTGTATTTGGTGTC	172
ISG15	ACAGCCATGGGCTGGGA	CTCTGACACCGACATGGAGC	87
NIBAN1	GCCGTCGGATCAACCTCC	AGCCTCAGTTTTCCCTCGGA	148
TMEM259	GCTCCATCAAGTTTGAGCTGGAC	TCCTGCGGCCACACTTTG	124
TMX4	AAAGGATGGGATATTCCGCCG	ACCAGCCATTCCAGACATCGTTA	145
ZFP28	AATGGCCTCGCATCCAAAGG	GTCACCAAGCCCTGGGACA	161

Table S2. Primary antibodies used for proteomics data validation.

Primary antibody	Antibody isotype	Source	Dilution
BACH	Mouse	Santa Cruz (sc-376808)	1:500
ERO1A	Mouse	Santa Cruz (sc-100805)	1:500
ESYT1	Mouse	Santa Cruz (sc-514488)	1:500
FINC	Mouse	Santa Cruz (sc-8422)	1:500
GARS	Mouse	Santa Cruz (sc-365311)	1:500
RAN	Mouse	Santa Cruz (sc-271376)	1:500
RPN1	Rabbit	Proteintech (12894-1-AP)	1:500
SERPH	Mouse	Santa Cruz (sc-5293)	1:500
UBP14	Mouse	Santa Cruz (sc-100630)	1:1000
VPS35	Mouse	Santa Cruz (sc-374372)	1:500
