# Supplementary figures and images for: Mutation-class dependent signatures outweigh disease-associated processes in cystic fibrosis cells
Source: Cell Biosci. 2023 Feb 9;13:26. doi: 10.1186/s13578-023-00975-y (PMC9912517; doi:10.1186/s13578-023-00975-y)

A

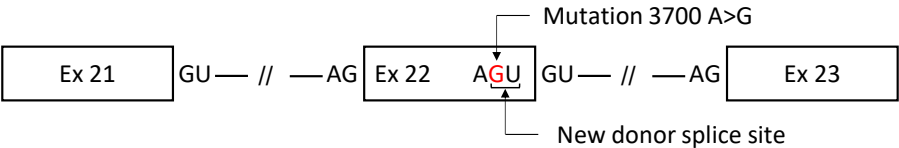

B

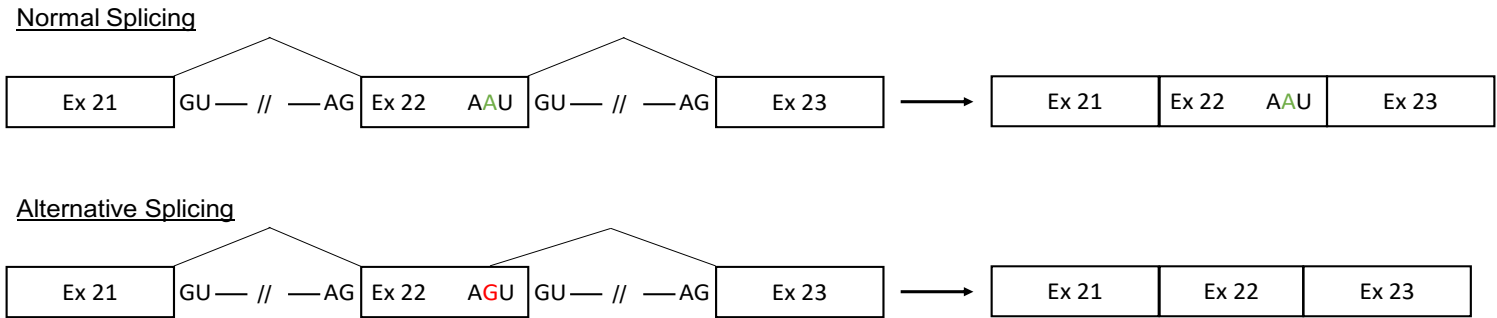

C

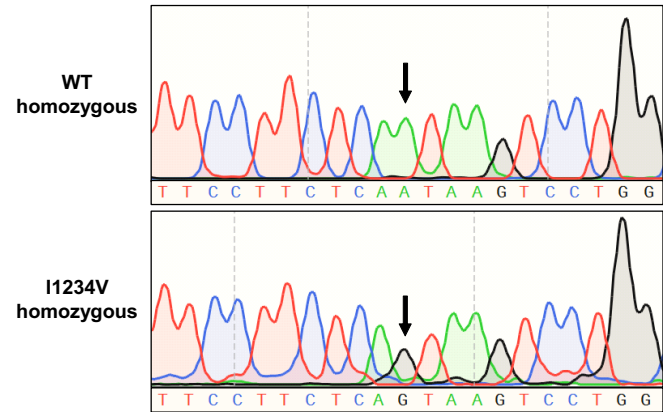

D

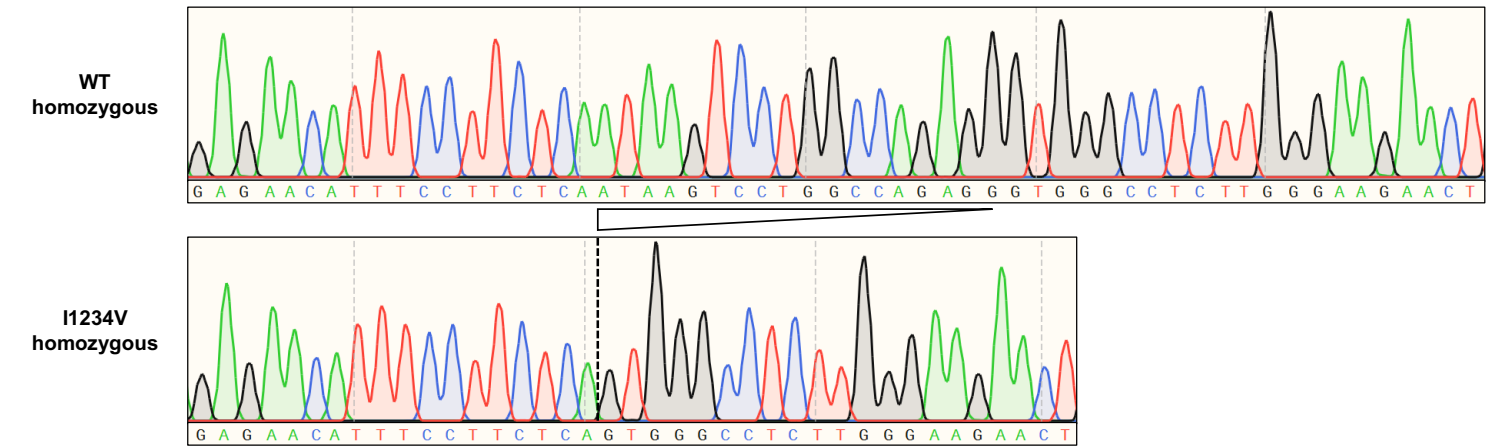

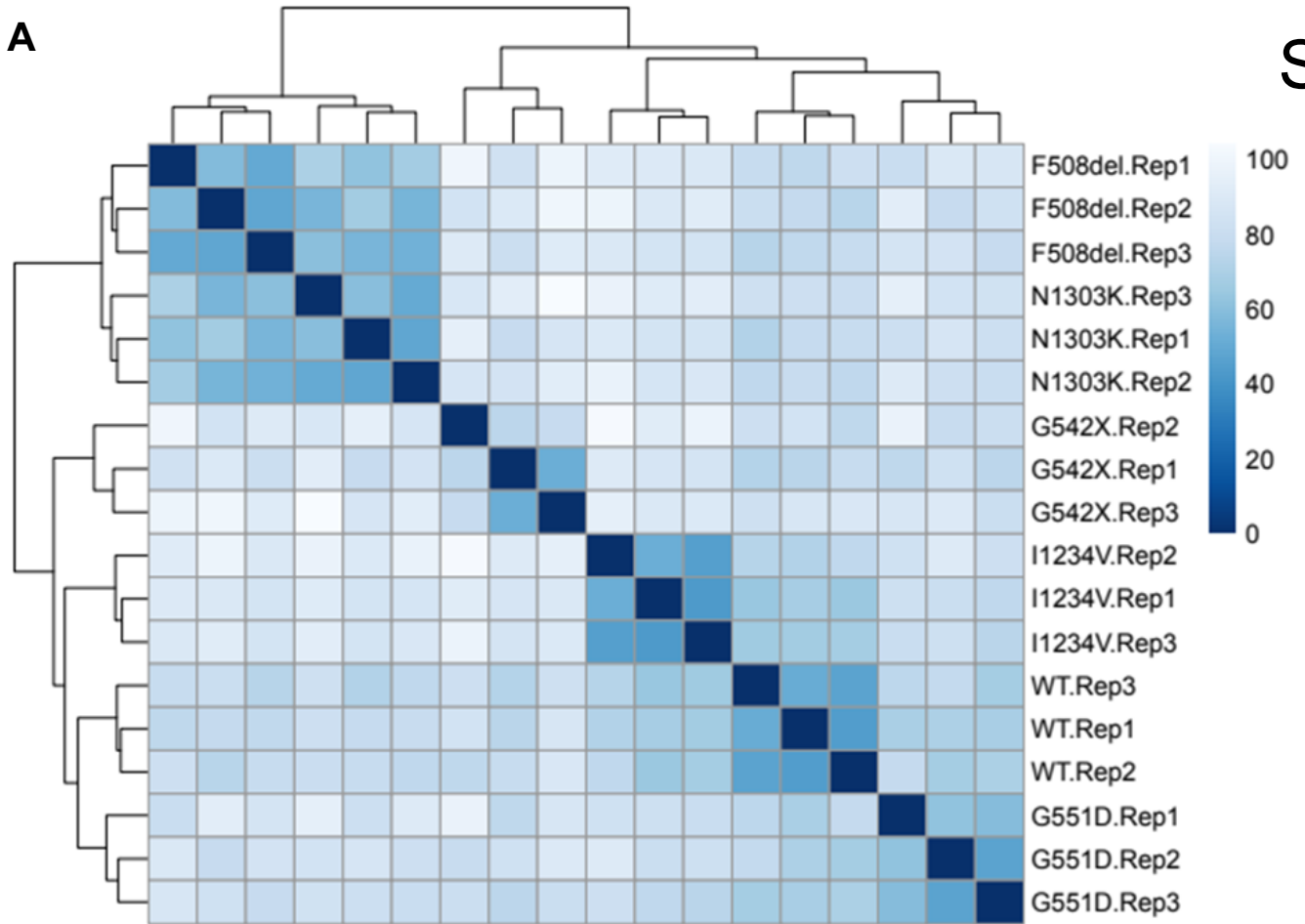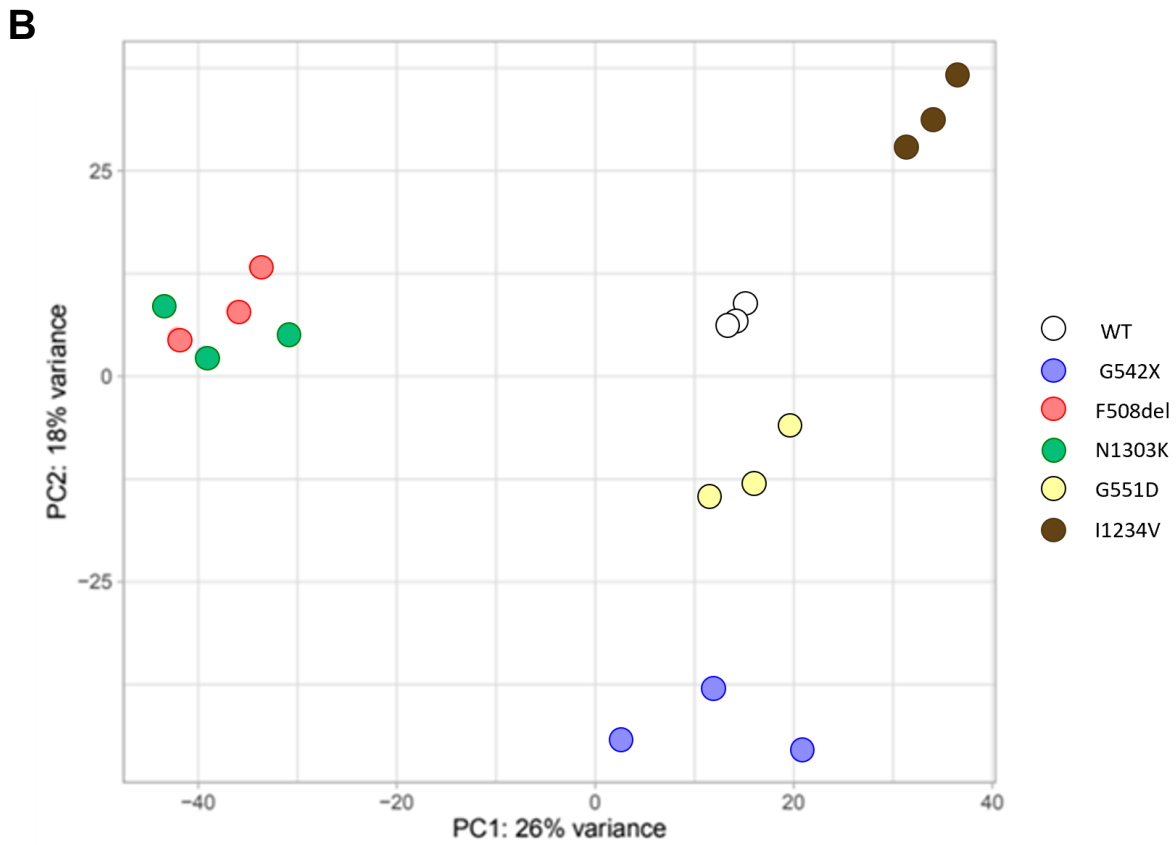

**A**

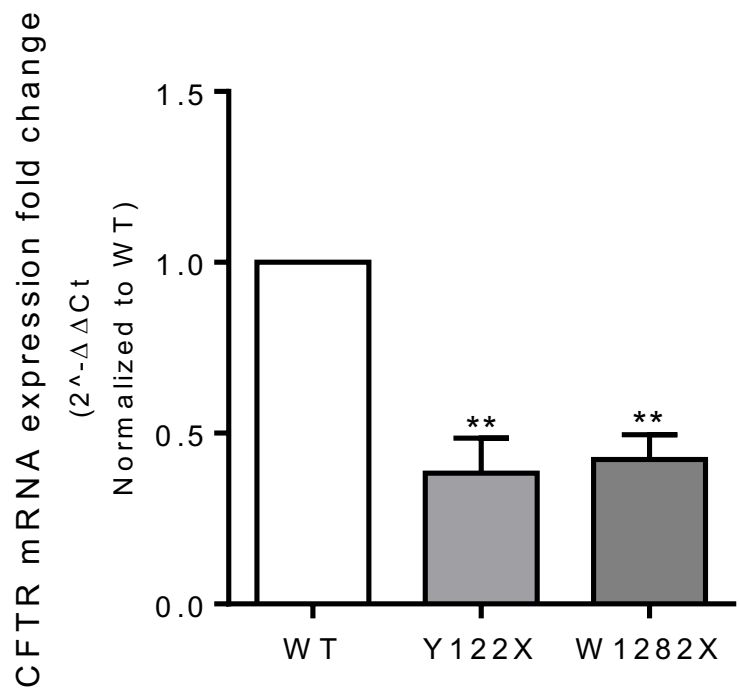

**B**

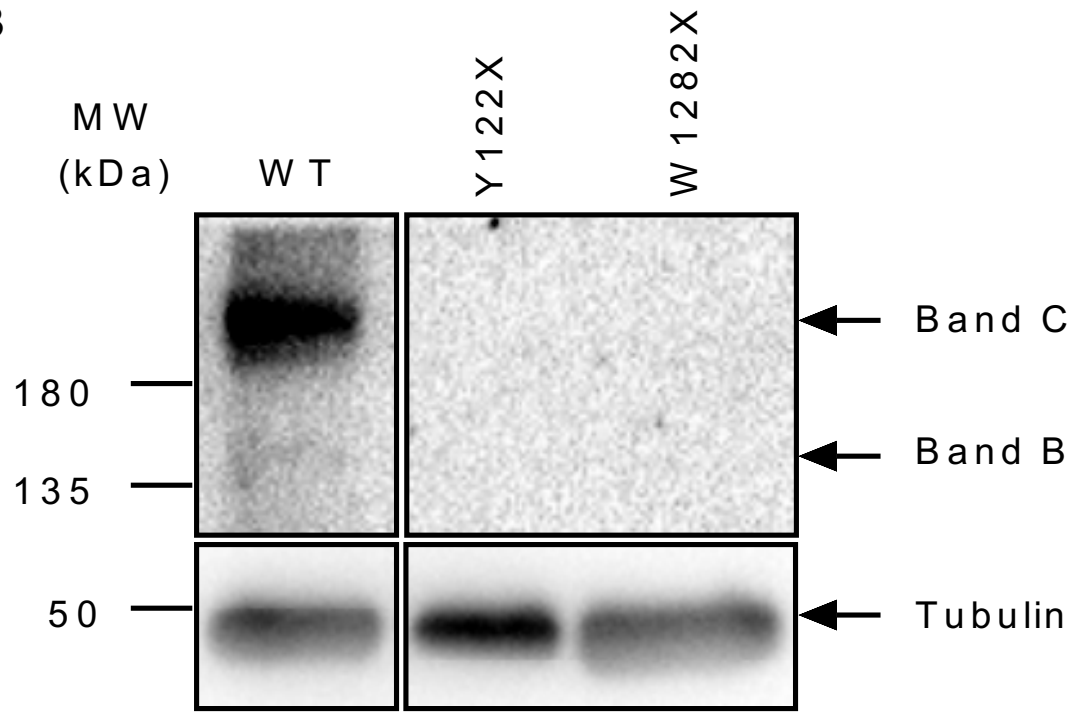

**A**

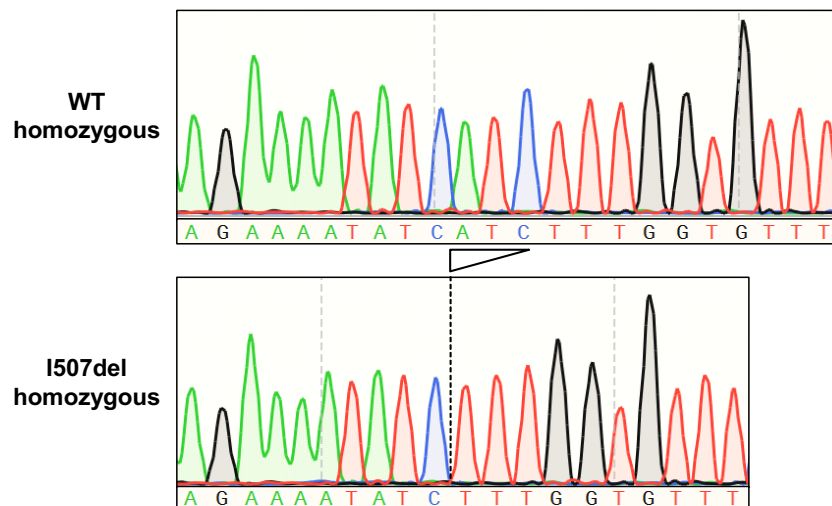

**B**

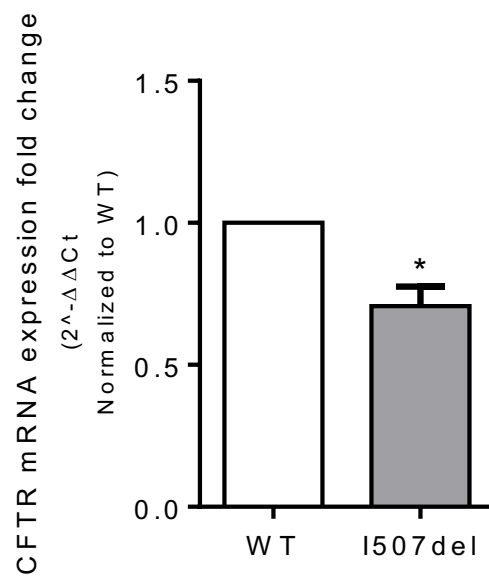

**C**

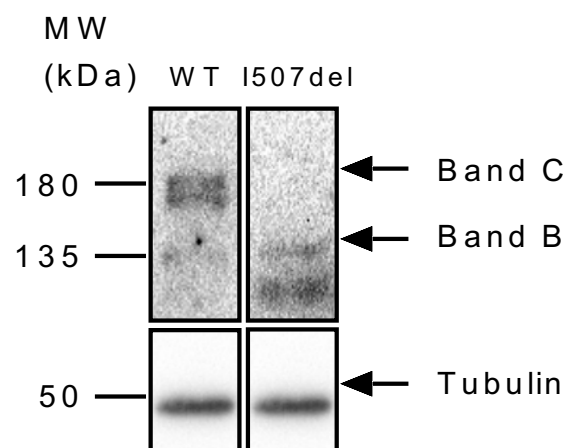

A

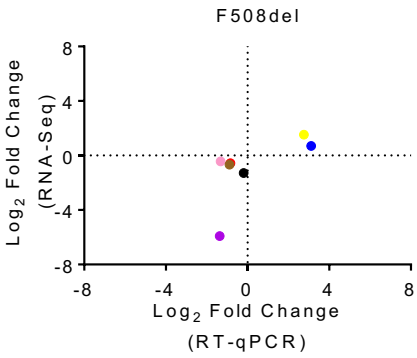

B

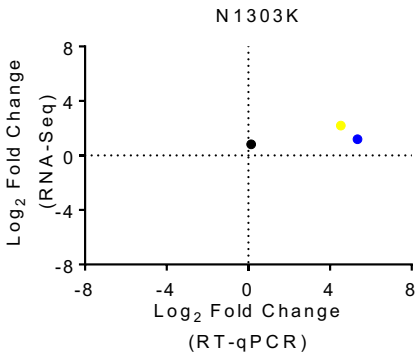

Legend:

- ARHGAP45
- ATP5D
- IFI44
- IFIT1
- NIBAN1
- TMEM259
- TMX4

C

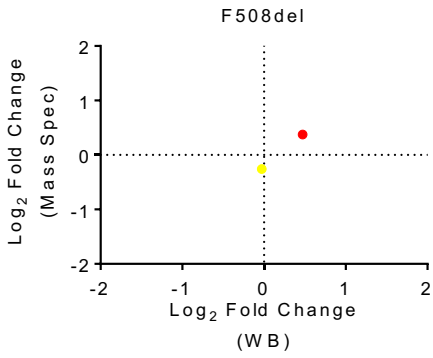

D

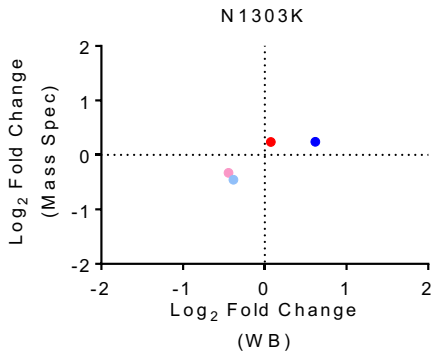

Legend:

- ESYT1
- FN1
- GARS1
- SERPINH1
- UB14

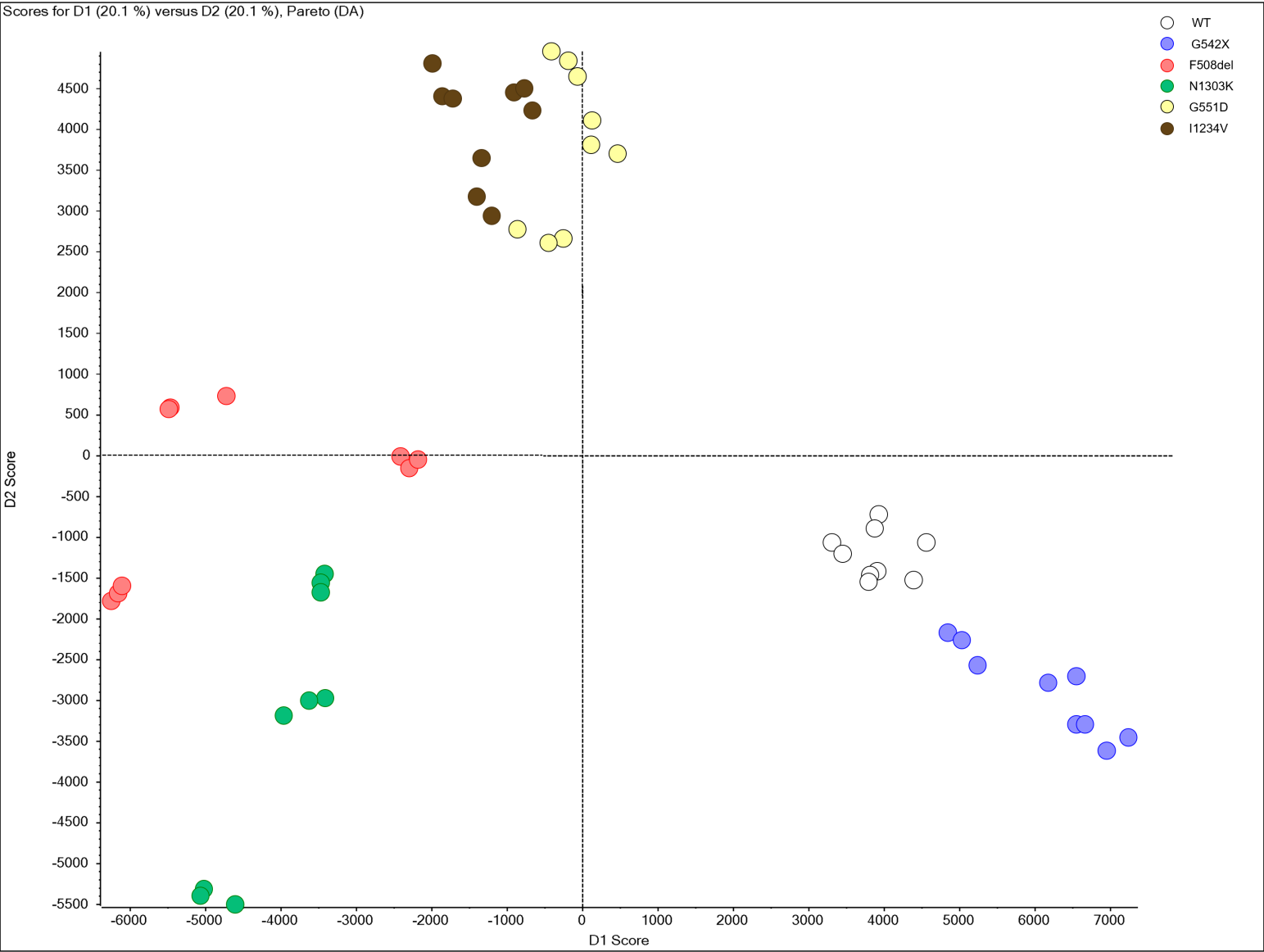

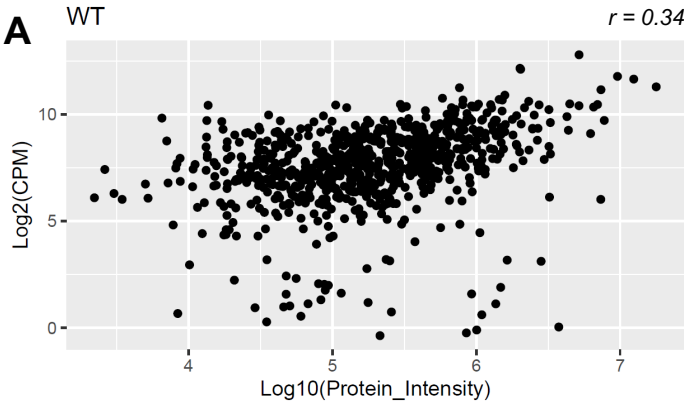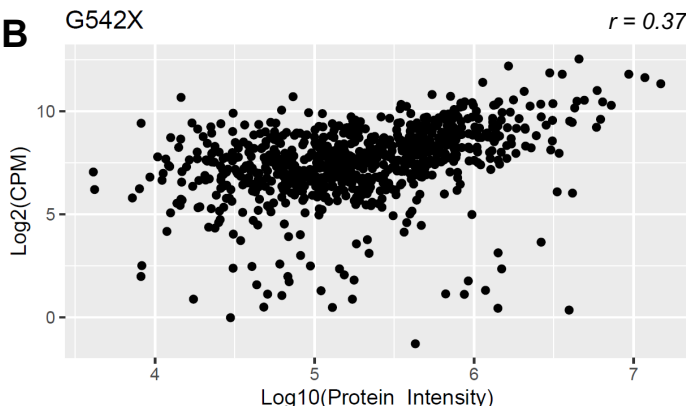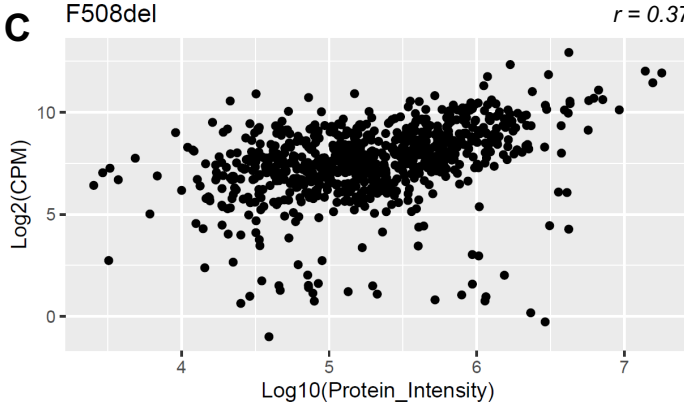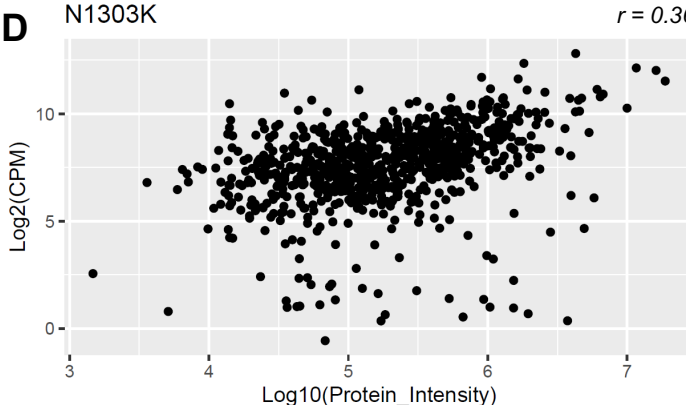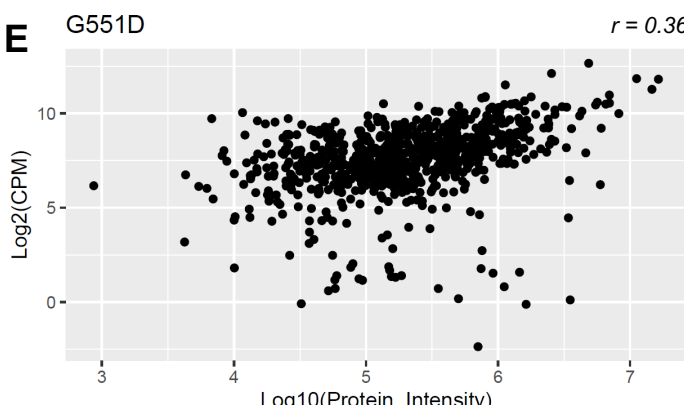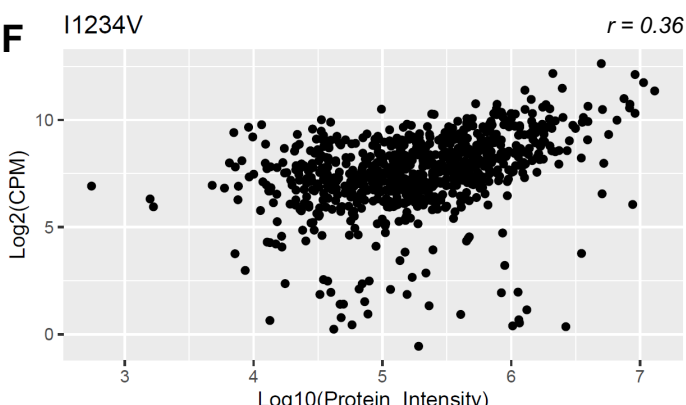

Supplement: Supplementary file 2 — Additional file 2: Figure S1. Characterization of the 16HBE I1234V-CFTR cell line A) The A > G alteration at position 3700 showed in red results in the creation of a cryptic donor splice site (GU). B) Scheme of splicing possibilities that occur in the presence of A (in green, WT) or a G (in red, I1234V) at position 3700 corresponding to normal or alternative splicing, respectively. C) Sequence of the WT cells (top panel) and the 16HBE I1234V-CFTR cell line (bottom panel). D) Sequence of the CFTR cDNA in the WT cells (top panel) and I1234V-CFTR cells (bottom panel) showing the aberrant transcript lacking 18-nt. Figure S2. Quality of the transcriptomics data A) Correlation of RNA-Seq data between each CFTR mutation replicate. B) Principal component analysis of the normalized RNA-Seq data for each genotype. Figure S3. Characterization of the CFF 16HBEge CFTR Y122X and CFF 16HBEge CFTR W1282X generated by the CFF Labs A) CFTR mRNA abundance normalized to GAPDH (house-keeping gene). Fold-change values are mean ± SEM relative to WT (n = 3 biological replicates). Vs. WT: **P ≤ 0.01. B) Western blot analysis of CFTR (UNC596) and Tubulin loading control for WT-CFTR, Y122X-CFTR, and W1282X-CFTR. Figure S4. Characterization of the 16HBE I507del-CFTR cell line A) Sequence of the 16HBE WT-CFTR (top panel) and the I507del-CFTR (bottom panel) cell lines confirming the genotype. B) CFTR mRNA abundance normalized to GAPDH (housekeeping gene). Fold-change values are mean ± SEM relative to WT (n = 3 biological replicates). Vs. WT: *P ≤ 0.05. C) Western blot analysis of CFTR (UNC596) and Tubulin loading control for WT-CFTR and I507del-CFTR cell lines. Figure S5. Validation of DEGs and DEPs in primary human nasal epithelial (pHNE) cells. DEGs common to all mutant cell lines were validated by RT-qPCR in A) F508del-CFTR and B) N1303K-CFTR cells. The 2-ΔΔCT method was used for data analysis using GAPDH as a housekeeping gene. DEPs common to all mutant cell lines were validated by WB in C) [file 13578_2023_975_MOESM2_ESM.pdf]
